# Genomic approaches in the search for molecular biomarkers in chronic kidney disease

**DOI:** 10.1186/s12967-018-1664-7

**Published:** 2018-10-25

**Authors:** M. Cañadas-Garre, K. Anderson, J. McGoldrick, A. P. Maxwell, A. J. McKnight

**Affiliations:** 1 Epidemiology and Public Health Research Group, Centre for Public Health, Belfast City Hospital, Queen’s University of Belfast, c/o University Floor, Level A, Tower Block, Lisburn Road, Belfast, BT9 7AB Northern Ireland UK; 2 Regional Nephrology Unit, Belfast City Hospital, Belfast, UK

**Keywords:** Genomic biomarkers, Genomics, Epigenetics, Transcriptomics, Chronic kidney disease, Diabetic kidney disease

## Abstract

**Background:**

Chronic kidney disease (CKD) is recognised as a global public health problem, more prevalent in older persons and associated with multiple co-morbidities. Diabetes mellitus and hypertension are common aetiologies for CKD, but IgA glomerulonephritis, membranous glomerulonephritis, lupus nephritis and autosomal dominant polycystic kidney disease are also common causes of CKD.

**Main body:**

Conventional biomarkers for CKD involving the use of estimated glomerular filtration rate (eGFR) derived from four variables (serum creatinine, age, gender and ethnicity) are recommended by clinical guidelines for the evaluation, classification, and stratification of CKD. However, these clinical biomarkers present some limitations, especially for early stages of CKD, elderly individuals, extreme body mass index values (serum creatinine), or are influenced by inflammation, steroid treatment and thyroid dysfunction (serum cystatin C). There is therefore a need to identify additional non-invasive biomarkers that are useful in clinical practice to help improve CKD diagnosis, inform prognosis and guide therapeutic management.

**Conclusion:**

CKD is a multifactorial disease with associated genetic and environmental risk factors. Hence, many studies have employed genetic, epigenetic and transcriptomic approaches to identify biomarkers for kidney disease. In this review, we have summarised the most important studies in humans investigating genomic biomarkers for CKD in the last decade. Several genes, including *UMOD*, *SHROOM3* and *ELMO1* have been strongly associated with renal diseases, and some of their traits, such as eGFR and serum creatinine. The role of epigenetic and transcriptomic biomarkers in CKD and related diseases is still unclear. The combination of multiple biomarkers into classifiers, including genomic, and/or epigenomic, may give a more complete picture of kidney diseases.

## Introduction

Chronic kidney disease (CKD) is recognised as a global public health problem [1] with adjusted CKD prevalence ranging between 3.3 and 17.3% in adult European populations [2]. CKD is more prevalent in older persons and is associated with multiple co-morbidities including an increased risk of cardiovascular disease (CVD). Diabetes mellitus (DM) and hypertension are common aetiologies for CKD. Other common causes of CKD include autoimmune renal diseases such as IgA glomerulonephritis (IgAN), membranous glomerulonephritis (MGN) and lupus nephritis (LN). Autosomal dominant polycystic kidney disease (ADPKD) is the commonest genetic disorder causing CKD [3].

The 2012 Kidney Disease: Improving Global Outcomes (KDIGO) Clinical Practice Guideline for the Evaluation and Management of CKD, developed by the Kidney Disease Outcomes Quality Initiative (KDOQI) of the National Kidney Foundation (NKF), recommends the use of estimated glomerular filtration rate (eGFR) in the evaluation, classification, and stratification of CKD [3]. The guidelines, categorise CKD into five stages based on eGFR measurements (Table 1) [3, 4]. The most widely used eGFR equation is derived from four variables (serum creatinine (SCr), age, gender and ethnicity), and is recommended by the KDOQI guidelines for initial assessment of kidney function [4].

**Table 1:** Categorisation of chronic kidney disease according to estimated glomerular filtration rate [4]

Chronic kidney disease group	eGFR (ml/min per 1.73 m^2^)	Terms
Group 1	≥ 90	Normal or high
Group 2	60–89	Mildly decreased
Group 3a	45–59	Mildly to moderately decreased
Group 3b	30–44	Moderately to severely decreased
Group 4	15–29	Severely decreased
Group 5	< 15	Kidney failure

*eGFR* estimated glomerular filtration rate

Although SCr assays are routinely available in clinical practice there are some limitations to its use as creatinine is influenced by muscle mass, exercise, age, gender, and ethnicity. Furthermore, ~ 50% of kidney function can be lost before the SCr rises above the normal laboratory range [5, 6]. An eGFR equation, based on SCr (eGFRcrea), is not an accurate measure for early stages of CKD, in elderly individuals with low muscle mass and in those with extreme body mass index values [7]. A second equation, based on the measurement of serum cystatin C, has been proposed as an alternative [4]. The use of eGFR based on cystatin C (eGFRcys) is recommended by the KDOQI guidelines as a confirmatory test to diagnose CKD in those specific circumstances when eGFR-creatinine is less accurate [3, 4]. However, serum cystatin C measurements also have recognised limitations since the circulating cystatin C level can be increased by inflammation, steroid treatment and thyroid dysfunction [8]. Urinary albumin, the principal component of urinary protein in most kidney diseases, is also routinely used as a marker of kidney damage. Although albuminuria may be an early sign of kidney disease in glomerulonephritis (an can occur prior to a decrease in glomerular filtration rate) it is not a universal feature of CKD [3]. Repeated assessments, employing the existing eGFR equations and urinary albumin measurements, can help to identify persons with CKD. There is however an ongoing need to identify additional non-invasive biomarkers that are useful in clinical practice to help improve CKD diagnosis, inform prognosis and guide therapeutic management.

CKD is a multifactorial disease with associated genetic and environmental risk factors. The increasing need to identify CKD patients at earlier stages and improve stratification of their risk for progression to end-stage renal disease (ESRD) has prompted many studies of novel and existing biomarkers for kidney disease in large cohorts of patients. Various approaches have been employed, from candidate single gene studies to genome wide multi-omic studies. Among them, many genetic, epigenetic, and transcriptomic studies have been developed in the hope that the introduction of novel technologies and unbiased approaches would allow the identification of new biomarkers for CKD that would also contribute to biological understanding of renal disease.

In this review, we will summarise the most important studies in humans investigating genomic biomarkers for CKD in the last decade.

## Genetic biomarkers

A genetic susceptibility to CKD exists although there is limited evidence for the individual gene variants responsible for this risk across the multiple aetiologies of CKD [9, 10]. Unlike Mendelian kidney disorders, such as ADPKD, where the causal variants are in the *PKD1* and *PKD2* genes [11], CKD is considered to be a polygenic disease in which many common, low-penetrance variants influence disease development and progression [12]. In the past, studies of candidate genes from pathways associated with renal function and linkage analysis in extended family pedigrees with multiple affected generations have been utilised to identify causal variants which contribute to CKD [13, 14]. However, both approaches have their drawbacks; candidate gene studies require knowledge of disease pathways and bias is introduced in gene selection, while genome-wide linkage analyses do not reflect genomic variation across all populations, as analyses are carried out within families [15]. More recently, genome-wide association studies (GWAS) have been employed to detect genetic variations in large numbers of unrelated individuals. For over 10 years, GWAS have been employed to identify single nucleotide polymorphisms (SNPs) associated with CKD and/or a range of traits associated with renal function. Serum creatinine (SCr), eGFR and urinary albumin-to-creatinine ratio (UACR), have been investigated using GWAS in different aetiologies of CKD and ESRD, such as diabetic kidney disease (DKD), MGN and IgAN. Genetic variants in over 50 loci have been associated with CKD or measures of renal function in different populations. Several of these gene variants are implicated in pathways driving the pathogenesis of CKD. The relevance of GWAS for the identification of SNPs associated with kidney transplantation outcomes and their potential use as genetic biomarkers for risk stratification or donor selection has been recently reviewed [16]. A small number of genetic variants were associated with graft function, T cell mediated rejection and tacrolimus trough levels, but these markers have not been validated in multiple different populations [16].

Various genetic loci have been associated with CKD (Table 2), but by far the most widely reported and consistently replicated genetic variants lie within the *UMOD* gene. *UMOD* codes for the protein uromodulin (previously known as Tamm-Horsfall [17], which is produced in the kidneys and is the most abundant urinary protein [18]. Loss of *UMOD* in mice leads to dysfunction of transmembrane solute transporters in the loop of Henle [19]. Mutations in *UMOD* have been associated with autosomal dominant tubulointerstitial kidney disease [20] which can progress to CKD [21]. Discovery analysis of 2388 CKD patients and 17,489 non-CKD controls and replication analysis of 1932 CKD patients and 19,534 controls of European ancestry, found *UMOD* variant rs12917707 to be significantly associated with both CKD and eGFR [22]. The same group confirmed these findings in a larger study in a total of 7173 European patients across the discovery and replication analyses [23]. A meta-analysis of population-based studies comprising over 130,600 individuals also found significant association with rs12917707 in 6271 cases of CKD (p = 3.7 × 10^−16^) and significant association in 2181 patients whose CKD was more severe (eGFR < 45 ml/min/1.73 m^2^; p = 1.1 × 10^−05^) [24]. An additional variant (rs4293393) in the *UMOD* gene was significantly associated with CKD (p = 4.1 × 10^−10^) in a study of 3203 Icelandic CKD patients and 38,782 controls [25]. This variant was replicated in a larger study of 15,594 Icelandic CKD patients (p = 9.1 × 10^−38^), in which another *UMOD* variant (rs11864909) was found to be significantly associated with CKD in a combined analysis (p = 2.2 × 10^−19^). Another *UMOD* variant, rs13329952 was significantly associated with CKD in a fixed-effects meta-analysis on a total of 151,137 individuals, of which 16,630 had CKD (p = 1.98 × 10^−25^); variant rs12917707 was also replicated in this meta-analysis (p = 1.16 × 10^−41^) [26].

**Table 2:** Genomic-wide association studies in chronic kidney disease

PMID	Author	Year	Patients	Ethnicity	Methodology	Main findings
28452372	Gorski	2017	110,517 eGFRcrea 24,063 eGFRcys	Caucasian	GWAMA 33 studies Imputation: 1KG 8,103,124 (SNPs and INDELs)	Identification of 49 genome-wide significant loci for eGFRcrea, including 10 novel loci All common variants except for one in *HOXD8* gene Confirmation of previously identified loci in or near *CST3/CST9* (p = 4.1·10^−153^), *UMOD* (p = 2.9·10^−10^), and *ATXN2* (p = 1.6·10^−8^) associated with eGFRcys Identification of 127 novel pathways for kidney function
27729571	Parsa	2017	CRIC cohort: 2807 CKD CKD definition: 20–70 ml/min/1.73 m^2^	1331 Black 1476 White	Illumina HumanOmni 1-Quad Array Platform	The discovery analysis identified 12 SNPs in black patients and 6 SNPs in white patients (p < 10^−6^) Followed up 3 SNPs in a replication cohort and 8 SNPs in a validation cohort In black, non-diabetic patients, variant rs653747 in LINC00923 was replicated (*p *= 0.039) in the African American Study of Kidney Disease and Hypertension Cohort and was also associated with ESRD (p = 4.90 × 10^−6^) Association of eGFR decline with rs931891 in the LINC00923 gene was found in white patients without diabetes (p = 1.44 × 10^−4^)
26831199	Pattaro	2016	Discovery: 49 population-based studies: 133,413 individuals Replication: 15 studies 42,166 individuals Trans-ethnic MA: 12 African studies 48 European studies	Caucasian African	GWAMA Discovery: ≈ 2.5million SNPs	The discovery analysis, stratified by diabetes status to account for its influence on CKD susceptibility, confirmed 29 previously identified loci and identified 18 new ones, 21 with genome-wide significance Overall, 43 SNPs were identified in association with eGFRcrea (nine in the non-diabetes sample), one with eGFRcys and four with CKD Replication analysis: 24 SNPs out of 48 novel candidates reached genome-wide significant (p < 5.0·10^−8^). Of these, 23 fulfilled additional replication criteria (q-value < 0.05) Suggested irrelevance of the estimation method for eGFR, since 22/24 retained significance across eGFR estimation method Association of 19 loci (13 demonstrated nominal associations) with eGFRcrea in diabetes The trans-ethnic MA showed that 12 loci (*SDCCAG8*, *LRP2*, *IGFBP5*, *SKIL*, *UNCX*, *KBTBD2*, *A1CF*, *KCNQ1*, *AP5B1*, *PTPRO*, *TP53INP2* and *BCAS1*) had fully consistent effect direction across the three ethnic groups For eGFRcrea, 15 out of 24 new loci were also genome-wide significant in the trans-ethnic MA
25082825	Sveinbjornsson	2014	81,656 and their 112,630 relatives 15,594 CKD (1716 Severe CKD) 291,420 non-CKD	Icelandic	Icelandic: Illumina, HumanHap300, HumanCNV370, HumanHap610, HumanHap1M, HumanHap660, Omni-1, Omni 2.5, Omni Express bead chips ≈ 24 million SNPs	Association of 19 SNPs with SCr (5 new) The genes affected by the novel variants encode either solute carriers (*SLC25A45*, *SLC47A1* and *SLC6A19*) or E3 ubiquitin ligases (*RNF128* and *RNF186*) Three of the five novel ones also associated with CKD (p < 0.05) No association with severe CKD, but consistent directional effect Replication of 41 out of 45 SNPs previously associated with SCr/CKD
24029420	Parsa	2013	CKDGen: Discovery: 26 cohorts 74,354 individuals Replication: 56,246 individuals	Caucasian	GWAMA	Identified OMIM genes associated with mendelian kidney disease and investigate SNPs within these for association with eGFR/CKD Identification of 8 independent associations with eGFR Confirmation of the previously identified variants rs12922822 in *UMOD* and rs12460876 in *SLC7A9* Three SNPs associated with eGFR (rs6433115 in *LRP2*, rs9827843 in *ROBO2*, and rs1050700 in *TSC1*) and one associated with CKD (rs249942 in *PALB2*) were taken to replication, with no significance achieved
22479191	Pattaro	2012	Discovery: 74,354: 6271 CKD 68,083 non-CKD 2181 CKD45 (eGFR < 45 ml/min/1.73 m^2^) 72,173 non-CKD45 Replication: 56,246 Ethnicity replication: 8110 (CARe)	Discovery and Replication: Caucasian Ethnicity replication: African American (CARe)	GWAMA	Twenty-one SNPs associated with eGFRcrea selected for replication (5 with genome-wide significance) Six SNPs showed genome-wide significance after replication: rs3925584 in *MPPED2* (overall); rs6431731 in *DDX1* (overall); rs2453580 in *SLC47A1* (non-diabetic); rs11078903 in *CDK12* (younger age); rs12124078 in *CASP9* (younger age); rs2928148 in *INO80* None of the six SNPs achieved significance in African American patients Similar results in eGFRcys Stratification by diabetes, hypertension, age and sex showed 29 SNPs (23 known and 6 novel ones) associated with eGFRcrea Stratifying by age showed *UMOD* associated with eGFRcrea in older individuals, and *CDK12* with younger Additional association of 18 out of 29 SNPs with CKD and 11 with CDK45
21355061	Böger	2011	GWAMA and discovery: CKDGen Consortium (20 studies) GWAMA: 31,580 individuals Discovery: 27,746 individuals Ethnicity replication: CARe Consortium 19,499 White 6981 African American	Caucasian African American	GWAMA and discovery: GWAMA Ethnicity replication: IBC SNP Chip Survival analysis: Illumina 1 M SNP chip	No genome-wide association (p < 5.0·10^−8^) with either UACR or microalbuminuria in both the overall and the nondiabetic analyses Top 16 independent SNPs taken forward to replication The combined analysis showed in direction-consistent association of rs1801239 in the *CUBN* gene with UACR (p = 4.0·10^−8^) UACR was also associated with rs17319721 in *SHROOM3*, whose minor allele (A) indicated lower albuminuria levels No association with eGFR (p = 0.53) or CKD (p = 0.33) was shown
20466664	Bostrom	2010	Discovery & Replication: 317 ESRD 354 non-nephropathy	African American	MassARRAY and DNA sequencing	No association with ESRD Three SNPs in intron 1 of *KL* associated with age of ESRD onset (association lost after Bonferroni correction)
20532800	Bostrom	2010	Discovery: non-DM ESRD (n = 500), pools Replication: same cohort, unpooled (n = 464) Controls: Non-nephropathy (Discovery = 500, Replication = 478)	African American	Illumina HumanHap550-Duo BeadChip and Affymetrix 6.0 Discovery: 166,033 SNPs Replication: 65 SNPs	Association of 16 SNPs with non-DM ESRD. Twelve out of 16 were located in/near the *MYH9* gene Four out 16 SNPs were not replicated Approximately 4% of carriers of *MYH9* risk allele progressed to ESRD, whereas 57% of non-nephropathy controls carried at least one copy of the risk haplotype, suggesting additional environmental and/or genetic factors contributing to the increased risk for ESRD in this population
20383145	Chambers	2010	Discovery: 23,812 individuals Replication: 16,626 individuals	Caucasian	GWAMA	Association of 109 SNPs at p < 5·10^−7^ level in the discovery stage Four SNPs (rs10206899, rs3127573, rs8068318, rs4805834) showed strong replication with creatinine concentration The variants rs10206899 (close to *NAT8*) and rs4805834 (close to *SLC7A9*) were associated with SCr, eGFR, cystatin-c and CKD The variants rs3127573 (near *SLC22A2*) and rs8068318 (located in *TBX2*) were only associated with SCr and eGFR None were associated with other clinical parameters which are known to influence SCr No association with *MYH9*
20686651	Gudbjartsson	2010	Discovery: 2903 CKD 35,818 Non-CKD controls Serum Creatinine: 22,256 individuals Replication: 300 CKD, 2964 Non-CKD controls SCr: 4198 Individuals	Discovery: Icelandic Serum Creatinine: 22,256 Icelandic Replication: 300 Icelandic (CKD) 2964 Icelandic SCr: 1819 Dutch 2379 Icelandic	Illumina HumanHap300 HumanHapCNV370 bead chips ≈ 2.5 million SNPs	Association of rs4293393-T in *UMOD* with increased risk of CKD (OR = 1.25; 95%CI 1.16–1.34; p = 6.2·10^−9^) and elevated SCr This association was replicated consistently No interaction of *UMOD*-rs4293393 with sex, but SCr increased 0.09 µmol/l per year in carriers of the T-allele (95%CI 0.07–0.11) Similar interaction of *UMOD*-rs4293393 and age with CKD The effect of *UMOD*-rs4293393 on SCr also increased with the number of comorbidities *UMOD*-rs4293393T was also associated with serum urea (p = 1.0 × 10^−6^) and uric acid concentrations (p = 0.0064), and was associated with a lower risk of kidney stones (OR = 0.88; p = 0.0053)
20383146	Kottgen	2010	Discovery: CKDGen Consortium (20 studies) 67,093 (5807 CKD) Replication: 22,982 (1366 CKD)	Caucasian	GWAMA	Association of 20 novel loci with eGFR and CKD. Thirteen loci were likely linked to renal function/CKD, others to creatinine production These variants accounted for 1.4% of variation in eGFR Most of the SNPs (65%) in the analyses were located in or within 3.7 kb upstream of genes Pathway analysis identified genes involved in nephrogenesis, glomerular function, podocyte function, solute transport, angiogenesis, or metabolic kidney function
19430482	Köttgen	2009	Discovery: 2388 CKD 17,489 non-CKD Cohorts: ARIC, CHS, FHS, RS Replication: 1932 CKD 19,534 non-CKD Cohorts: AGES WGHS	European	GWAMA: 300,000–900,000 SNPs	SNPs with suggestive (p < 4·10^−7^) or significant (p < 5·10^−8^) genome-wide associations identified in the discovery were tested for in silico replication All the significant loci from the discovery analysis replicated for eGFRcrea except for the intronic SNP rs6040055 in *JAG1* on chromosome 20 (p-overall = 0.006) Variants in 5 different genes (*UMOD, SHROOM3, SPATA5L1/GATM, STC1 and CST3/CST9*) showed significant association (p < 5 × 10^−8^) with CKD, eGFRcrea or eGFRcys, with several variants showing association with more than one of these traits
18522750	Kottgen	2008	ARIC Cohort: 15,111	11,217 White 3894 Black	iPLEX gold assay Taq-Man: 16 SNPs	The intronic SNP rs6495446 in the *MTHFS* gene was significantly associated with CKD among white ARIC participants (p = 0.001)
17903292	Hwang	2007	FHS cohort: 1345 individuals 1010 eGFR: 981 Cystatin-C 822 UAE	Caucasian	Affymetrix GeneChip Human Mapping 100 K (70,987 SNPs)	Identified several SNPs associated with each phenotype The top SNP associated with each trait was listed: eGFR (rs2829235, p = 1.6 × 10^−05^), Cystatin-C (rs1158167, p = 8.5 × 10^−09^) which is found near the cystatin-C precursor gene family (CST3, CST4, CST9), and UAE (rs1712790, p = 1.9 × 10^−06^) in *NXPE2*

1KG: 1000 genomes (1KG) reference panel; A1CF: APOBEC1 complementation factor; AGES: Age Gene/Environment Susceptibility-Reykjavik Study; AP5B1: adaptor related protein complex 5 subunit beta 1; ARIC: Atherosclerosis in Communities; BCAS1: breast carcinoma amplified sequence 1; CARe: Candidate-gene Association Resource Consortium; CASP9: caspase 9; CDK12: cyclin dependent kinase 12; CHS: Cardiovascular Health Study; CI: Confidence interval; CKD: Chronic Kidney Disease; CKDGen: CKDGen Consortium;CRIC: Chronic Renal Insufficiency Cohort; CST3: cystatin C; CST4: cystatin S; CST9: cystatin 9; CUBN: cubilin; DDX1: DEAD-box helicase 1; DM: Diabetes mellitus; eGFR: estimated glomerular filtration rate; eGFRcrea: GFR estimated using serum creatinine concentration; eGFRcys: GFR estimated using serum cystatin-c concentration; ESRD: end-stage renal disease; FHS: Framingham Heart Study; GATM: glycine amidinotransferase; GWAMA: genome-wide association meta-analysis; HOXD8: homeobox D8; IBC: ITMAT/Broad/CARE Vascular Disease 50 k (IBC) single-nucleotide polymorphism (SNP) chip arrayI; GFBP5: insulin like growth factor binding protein 5; INDEL(s): insertion/deletion(s); INO80: INO80 complex subunit; JAG1: jagged 1; KBTBD2: kelch repeat and BTB domain containing 2; KCNQ1: potassium voltage-gated channel subfamily Q member 1; KL: klotho; LINC00923: long intergenic non-protein coding RNA 923; LRP2: LDL receptor related protein 2; MA: meta-analysis; MPPED2: metallophosphoesterase domain containing 2; MTHFS: methenyltetrahydrofolate synthetase; MYH9: myosin heavy chain 9; NAT8: N-acetyltransferase 8 (putative); NXPE2: neurexophilin and PC-esterase domain family member 2; OMIM: Online Mendelian Inheritance in Man; OR: Odds ratio; PALB2: partner and localizer of BRCA2; PTPRO: protein tyrosine phosphatase, receptor type O; RNF128: ring finger protein 128; E3 ubiquitin protein ligase; RNF186: ring finger protein 186; ROBO2: roundabout guidance receptor 2; RS: Rotterdam Study; SDCCAG8: serologically defined colon cancer antigen 8; SCr: Serum creatinine; SHROOM3: shroom family member 3; SKIL: SKI like proto-oncogene; SLC22A2: solute carrier family 22 member 2; SLC25A45: solute carrier family 25 member 45; SLC47A1: solute carrier family 47 member 1; SLC6A19: solute carrier family 6 member 19; SLC7A9: solute carrier family 7 member 9; SPATA5L1: spermatogenesis associated 5 like 1; SNP: single nucleotide polymorphism; STC1: stanniocalcin 1; TBX2: T-box 2; TP53INP2: tumor protein p53 inducible nuclear protein 2; TSC1: TSC complex subunit 1; UACR: Urinary albumin-to-creatinine ratio; UNCX: UNC homeobox; WGHS: Women’s Genome Health Study

There are ethnic differences in the incidence and prevalence of CKD; the incidence of ESRD is almost five times higher in African Americans than Americans of European descent [27]. A study of 1372 African American ESRD patients and 806 controls identified several variants in the myosin heavy chain type II isoform A (*MYH9*) gene as being specifically associated with non-diabetic ESRD in African Americans [28]. To further investigate this association, ESRD patients were sub-divided by presence of diabetes. No association was found in diabetic ESRD patients, but significance of *MYH9* variants was retained for the non-diabetic ESRD group [28]. Similar association was found in variants spanning exon 14–23 of the *MYH9* gene in 852 focal segmental glomerulosclerosis (FSGS) and 433 non-diabetic ESRD patients, but not for 476 diabetic-ESRD patients when compared to 222 controls [29]. The results for ESRD patients were consistent with the first analysis, and both studies described a specific association between variant rs735853 and non-diabetic ESRD patients, but not with diabetic ESRD patients [28, 29]. A study of 464 non-diabetic ESRD patients and 478 controls, replicated in 336 non-DM ESRD cases and 363 controls, identified 16 SNPs associated with non-diabetic ESRD, 12 of which were found in/near *MYH9* [30]. To reinforce the specificity of these variants in African American individuals, a sufficiently powered study in 23,812 European patients found no association of *MYH9* with eGFR or CKD [31]. Despite the lack of previous association with diabetic-ESRD, two subsequent studies found significant association of *MYH9* with type 2 diabetes mellitus (T2DM)-ESRD [32, 33]. The *MYH9* gene codes for myosin non-muscle myosin heavy chain IIA protein [34], a protein involved in cytokinesis, chemotaxis and differentiation of non-muscle cells [35]. Mutations in this gene are responsible for a number of autosomal dominant conditions and patients, such as those with Epstein Syndrome, can develop nephritis or other renal abnormalities [36]. This known link to renal function further supports the role of *MYH9* as a genomic biomarker in CKD, although further studies in patients of European descent with CKD may be required to determine its suitability a biomarker across populations or should be limited to African Americans.

However, strong linkage disequilibrium found among variants in *MYH9* and the adjacent *APOL1* gene made it unclear as to which variants were associated disease [37]. With the release of data from the 1000 genomes project, two independent groups identified variants in *APOL1* which were associated with non-diabetic ESRD [38, 39]. In a study of 1030 non-diabetic ESRD patients and 1025 non-diabetic non-ESRD controls, two variants in *APOL1* (rs73885319 and rs71785313, referred to as G1 and G2, respectively) reached genome-wide significance (G1: p = 1.1 × 10^−39^, G2: p = 8.8 × 10^−18^) [39]. The same study found similar association of G1 (p = 1.07 × 10^−23^) and G2 (p = 4.38 × 10^−7^) with FSGS in 205 FSGS African Americans patients and 180 non-FSGS controls [39]. After adjusting for G1 and G2 using logistic regression, association with *MYH9* was lost for both non-diabetic ESRD and FSGS, but association with variants in *APOL1* remained when adjusting for variants in *MYH9* [39]. An independent group was carrying out similar analysis at the same time in 346 African American and 84 Hispanic American ESRD patients and 147 African American and 378 Hispanic American controls [38]. In this study they also found that variants in *APOL1* (rs73885319 and rs60910145) were much more strongly associated with ESRD than all previously reported variants in *MYH9* [38]. The G1 and G2 risk alleles were also found to be associated with a greater risk of developing CKD (OR = 1.49, CI 1.02–2.07) and greater risk of progression to ESRD (OR = 1.88, CI 1.20–2.93) in 3067 African American individuals did not have CKD at baseline, of which 190 went on to develop CKD and 114 developed ESRD [40].

*APOL1* codes for apolipoprotein L1 (APOL1), a protein which interacts with high density lipoprotein (HDL) cholesterol in plasma [41]. *APOL1* was identified as a protective factor in human sleeping sickness, common in Sub-Saharan Africa [42], and it has been shown that only variants in *APOL1* linked with renal dysfunction confer protection from disease [39]. Therefore, these variants may have been passed on due to their protective effect against human sleeping sickness in Africans, resulting in higher prevalence of *APOL1* variants in individuals of African origin, compared to those of Europeans.

Other loci have shown association with CKD, such as *PRKAG2* [23, 24, 26, 43] and *WDR37* [23, 24, 26, 43], variants within these genes are reported less often than those in UMOD or *MYH9/APOL1*.

### eGFR

Variants in the *UMOD* gene have demonstrated the ability not only to predict the risk of CKD, but also changes in eGFR. The rs12917707 variant was the first SNP in *UMOD* to reach genome-wide significance for both SCr eGFRcrea and eGFRcys [22]. Estimating GFR independently of creatinine discriminates between association derived from renal function and from creatinine production or metabolism. Meta-analysis of discovery (n = 19,877) and replication (n = 21,466) analyses of independent European patient cohorts showed significance of *UMOD* with eGFRcrea (p = 3.0T × 10^−11^) and eGFRcys (p = 2.0 × 10^−07^), showing *UMOD* is associated with renal function decline and not creatinine production or metabolism [22]. The rs12917707 variant has been associated with SCr/eGFR in patients of European ancestry in several other studies, both with and without diabetes [24, 44, 45]. It also showed a stronger association with older individuals when patient groups were stratified by age (*p *= 8.4 × 10^−13^) [24] and with significant changes in eGFR over time and kidney function decline in a total of 63,558 patients [45]. Another variant in *UMOD* (rs4293393) correlated with SCr in a combined analysis of 24,375 Icelandic and Dutch participants [25]. This variant was replicated for SCr in 194,286 Icelandic individuals (p = 2.48 × 10^−38^), where additional *UMOD* variants (rs11864909; p = 4.05 × 10^−21^) and rs12917707 (p = 2.03 × 10^−36^) also reached genome-wide significance [43]. More recently, rs12917707 and another *UMOD* variant (rs13329952, p = 9.47 × 10^−43^) were found to be significantly associated with eGFRcrea. Variant rs12917707 showed association both in patients with diabetes (n = 118,365, p = 2.48 × 10^−08^) and without (n = 11,522, p = 4.68 × 10^−36^) [26].

In summary, as several different *UMOD* SNPs have been linked to both CKD and eGFR, *UMOD* may prove to be useful as a diagnostic and prognostic genomic biomarker of CKD. Indeed, levels of serum uromodulin have recently been shown to useful in disease detection as CKD patients were shown to have a significantly lower serum uromodulin concentration (p < 0.001) and measurement of serum *UMOD* was shown to significantly enhance performance of a CKD prediction model (p = 0.049) [46]. *UMOD* often exhibits one of the strongest associations with CKD [22, 26], suggesting that *UMOD* would likely be suitable as a diagnostic genomic biomarker of CKD.

Multiple variants (rs17319721 [22, 24, 25, 43, 44], rs2137154 [43], rs9992101 [43] and rs13146355 [43] in *SHROOM3* have also been associated with eGFR in patients of European ancestry [22, 24, 25, 43, 44]. *SHROOM3* encodes the shroom3 protein, which has been shown to regulate cell shape by coordinating cytoskeleton assembly [47] and whose overexpression (due to presence of variant rs17319721) is associated with increased allograft fibrosis in renal transplant patients [48]. The *SHROOM3* rs17319721 variant was first identified in patients within four cohorts from Cohorts for Heart and Aging Research in Genomic Epidemiology (CHARGE) consortium [22]. Meta-analysis of 19,877 patients of European ancestry from CHARGE, to two additional population-based studies (n = 21,466) were used for discovery and replication, respectively [22]. After *UMOD*, the most significant genome-wide association with eGFR (estimated using SCr) was with *SHROOM3*, found in both discovery (p = 9.7 × 10^−08^) and combined discovery and replication meta-analyses (p = 1.2 × 10^−12^) [22]. The same variant showed association with SCr in a study of 22,256 Icelandic patients (*p*  =  0.00057) [25], and association with eGFR was observed in a combined discovery and replication meta-analysis of 74,354 and 56,346 patients of European ancestry (p = 1.5 × 10^−21^) [24]. In the same study, association of *SHROOM3* (rs17319721) and eGFR remained significant after stratifying patients by age, sex, diabetes and hypertension status, but a parallel investigation in patients of African ancestry yielded no association, highlighting the genetic differences between patients of different ethnic origins [24]. The rs17319721 variant in *SHROOM3* was associated with eGFR in a study of 3028 Caucasian patients with type 2 diabetes (p = 3.18 × 10^−03^), showing a larger impact on baseline eGFR when patients presented with albuminuria [44]. In a study of 81,656 Icelandic patients and their 112,630 relatives (correction made for relatedness), genome-wide significance was reached for each variant with SCr, in both discovery and replication analyses (rs17319721: p = 4.8 × 10^−10^, rs2137154: p = 4.7 × 10^−13^, rs9992101: p = 1.0 × 10^−10^ and rs13146355: p = 6.5 × 10^−12^) [43].

A number of other genes have shown specific association with eGFR, such as *STC1* [23–26, 43], *SLC22A2* [23, 24, 26, 31, 43] and *WDR37* [23, 24, 26, 43, 49] are thought to be involved in creatinine secretion, rather renal function, therefore not being representative of CKD. This highlights the importance of functional analysis of significantly associated variants to determine their role in CKD. A second measure of eGFR, such as serum cystatin-C, may need to be incorporated into future studies to control for variants associated with creatinine secretion.

### Diabetic kidney disease

The Engulfment and Cell Motility 1 gene (*ELMO1*) has been studied extensively in DKD (Table 3). Located on chromosome 7, *ELMO1* codes for a protein of the same name involved in a pathway promoting phagocytosis of apoptotic cells [50]. A variant in intron 18 of chromosome 7p14 showed significant association with DKD (p = 5 × 10^−5^) in 560 Japanese patients and 360 T2DM controls [51]. As DKD often leads to ESRD [52], a follow-up study of *ELMO1* gene variants in African American patients with T2DM-ESRD was carried out [53]. Combined analyses for a total of 1135 T2DM-ESRD patients was performed compared to a combination of healthy (n = 596), non-diabetic ESRD (n = 326) and T2DM controls (n = 328) in order to determine which SNPs were specifically associated with T2DM-ESRD and not the individual T2DM or ESRD traits [53]. A total of 13 variants in *ELMO1* showed significant association with T2DM-ESRD, 11 of which were found in intron 13 [53]. These variants showed no association in patients with T2DM or ESRD singularly, indicating these SNPs are specific for DKD [53]. To extrapolate these findings in Japanese and African American patients, an additional study was carried out in patients of European ancestry, to determine if association of *ELMO1* and DKD was independent of ethnic origin. In a study of advanced DKD, defined as the presence of persistent proteinuria or ESRD, 284 DKD patients with proteinuria and 536 patients with DKD-ESRD were analysed for association of 359,193 SNPs, 106 in *ELMO1,* versus 885 type 1 diabetes mellitus (T1DM) controls [54, 55]. Eleven nominally significant SNPs were identified within four novel genomic loci, including variants in *FRMD3* (p = 5.0 × 10^−7^) and *CARS (*p = 3.1 × 10^−6^), which were also shown to contribute significantly to time needed to develop DKD (*FRMD3,* p = 0.02 and *CARS,* p = 0.01) [54].

**Table 3:** Genomic-wide association studies in diabetic kidney disease

PMID	Author	Year	Patients	Ethnicity	Methodology	Main findings
25493955	Gorski	2015	63,558 individuals CKDGen: Stage 1 MA: 16 cohorts Stage 2 MA: 13 cohorts Rapid decline: (annual eGFR decline ≥ 3 ml/min/1.73 m^2^)	Caucasian	GWAMA	Association of rs12917707 at the *UMOD* locus with an increase in eGFR over time at a genome-wide significant level (p = 2.6·10^−14^) The novel *CDH23*, *GALNTL5/GALNT11*, *MEOX2*, *IL1RAP/OSTN*, *C2orf48/HPCAL1* and *NPPB/NPPA* loci were associated with CKD or eGFR at a significance level of p < 10^−6^ In stage 2 MA, only rs12917707 at *UMOD* was significantly associated with the stage 1 trait after correcting for multiple testing (p = 4.7·10^−5^) Two SNPs showed suggestive significance (one-sided p < 0.05) with their respective stage 1 trait: rs875860 in *CDH23* with eGFR change in those with CKD at baseline, and rs1019173 at *GALNTL5/GALNT11* with rapid decline in eGFR The combined stage 1 and stage 2 analysis showed genome-wide significant association only for *UMOD* (rs12917707, p = 1.2·10^ 16^) The combined analysis also showed suggestive evidence of association for the two novel loci identified in stage 1 (rs875860 in *CDH23*: p = 1.5·10^−6^ with eGFR change in CKD patients; *rs1019173* at GALNTL5/GALNT11: OR(A-allele) = 0.91, p = 2.2·10^−7^ with rapid decline in eGFR)
23586973	Deshmukh	2013	GoDARTS cohort 3028 T2DM Sustained normoalbuminuria: ACR < 2.5 mg/mmol (males), < 3.5mh/mmol (females)	Caucasian	Affymetrix Genome-Wide Human SNP Array 6.0 (16 SNPs)	Replication of the association of *UMOD*, *GCKR* and *SHROOM3* with eGFR in patients with T2DM, confirming the findings of previous studies No interaction of *UMOD* with age in patients with T2DM (p = 0.84) None of the 16 SNPs were associated with time to stage 3B CKD at the predefined threshold of 0.003. *UMOD* and *SLC7A9* were associated with time to stage 3B CKD at the threshold of 0.05 (consistent direction of effects with previous reports) Clear difference in the effect sizes in patients with sustained normalbuminuria and those with albuminuria for *UMOD* and *SLC7A9* genes. *UMOD* has twice the effect in patients with sustained normalbuminuria as compared with those with albuminuria (p-interaction = 0.002) *SHROOM3* (p-interaction = 0.003) and *GCKR* (p-interaction = 0.08) showed larger effect sizes in patients with albuminuria
23028342	Sandholm	2012	2916 T1DM (460 mAlb) 1399 T1DM-ESRD 5253 T1DM no kidney disease GENIE cohort: UK-ROI, FinnDiane, GoKinD Additional MA: 9 cohorts (n = 5873)	Caucasian	UK-ROI: Omni1-Quad array FinnDiane: Illumina’s BeadArray 610-Quad array GoKinD: Affymetrix 500 K (≈ 2.4 million SNPs)	In the MA, only association of rs7583877 with ESRD analysis reached genome-wide significance Five independent signals in total DKD and 6 other signals in ESRD anaylsis reached p < 10^−5^ In the combined meta-analysis with 41 SNPs and 9 additional cohorts, association of the intronic SNP rs7583877 in *AFF3* with ESRD retained genome-wide significance (OR = 1.29; 95%CI 1.18–1.40; p = 1.2·10^−8^ Another locus (rs12437854), located between the *RGMA* and *MCTP2* genes on chromosome 15q26 also reached genome-wide significance for association with ESRD (OR = 1.80; 95%CI 1.48–2.17; p = 2.0·10^−9^) The top SNP associated with the primary DKD phenotype identified from the combined discovery and second stage analysis was rs7588550, an intronic SNP in the *ERBB4* gene, which demonstrated consistent protective effects in the replication samples. However, this SNP did not reach genome-wide statistical significance (OR = 0.66; 95%CI 0.56–0.77; p = 2.1·10^−7^)
22721967	Williams	2012	903 UK-ROI 251 FinnDiane (n = 251; ESRD + laser) GENIE cohort: UK-ROI: 903 T1DM + DKD 1001 T1DM FinnDiane 251 T1DM + DKD 987 T1DM GoKinD	Caucasian	UK-ROI (791,687 SNPs): sequenom iPLEX TaqMan FinnDiane (549,530 SNPs): Illumina’s BeadArray, 610Quad array, TaqMan GoKinD (360,899 SNPs) Total tested: 2199	The rs1617640 variant in the *EPO* promoter showed same effect direction as seen in the original report Fixed effects MA showed no genome-wide significance for association of rs741301 in *ELMO1* with DKD. No association in individuals cohorts either
21150874	McDonough	2011	Discovery: 965 T2DM + ESRD, 1029 NDNN Replication: 709 T2DM + ESRD, 690 NDNN Trait GWAS: 1246 T2DM no kidney disease, 1216 non-diabetic ESRD	African American	Affymetrix 6.0 Chip Discovery: 832,357 SNPs	Identification of 126 SNPs associated with a level of p < 10^−4^ Top discovery hit was rs5750250 in *MYH9* gene Of the top 724 hits taken into replication analysis, 67 SNPs showed nominal association (p < 0.05) No genome-wide association SNPs for T2DM-ESRD Trait discrimination GWAS identified 25 SNPs specifically associated with T2DM-ESRD, as no association was seen in T2DM patients without nephropathy Association of *SASH1*, *RPS12*, *AUH*, *MSRB3*-*HMGA2* and *LIMK2*-*SFI1* with T2DM-ESRD Association of *LIMK2*-*SFI1* and *MYH9* with ESRD
19929986	Craig	2009	GoKinD cohort Discovery: 547 T1DM + persistent proteinuria or ESRD, 549 T1DM Replication: 462 T1DM + ESRD, 470 T1DM	Caucasian	Discovery: Infinium II HumanHap 550Beadchip 474,050 SNPs Replication: iPLEX assay 22 SNPs	Discovery: 2870 SNPs showing “substantial differences” in mean allele frequency (p < 0.05) Twenty-two SNPs included in the Replication found nominal association (p = 0.05)
19183347	Leak	2009	Discovery: 577 T2DM-ESRD, 596 NDNN Replication: 558 T2DM-ESRD, 328 T2DM, 326 non-diabetic ESRD	African American	Illumina Genotyping Services iPlex methodology on a MassARRAY genotyping system (311 SNPs)	Association of rs3778713, rs17171024 and rs1647791 in *ELMO1* with T2DM-ESRD, although not later replicated Replication of 20 SNPs out of the 98 included The combined analysis found 27 SNPs (11 located in intron 13) nominally associated with T2DM-ESRD None of these SNPs were associated with T2DM or ESRD as separate entities Other SNPs did associated with T2DM or ESRD individually
19252134	Pezzolesi	2009	Discovery: GoKinD Replication: DCCT/EDIC, 284 T1DM + Proteinuria, 536 T1DM + ESRD, 885 T1DM Replication: 1304 T1DM + persistent proteinuria or ESRD	Caucasian	Discovery: Affymetrix 5.0 500 K TaqMan 467,144 SNPs Replication: Illumina Human1M Beadchip 840,354 SNPs	No SNP achieved genome-wide significance in the discovery phase Identification of 11 SNPs from four distinct chromosomal regions with p < 10^−5^, taken for replication The rs10868025 variant on chromosome 9q showed the strongest association with DKD (OR = 1.45; p = 5.0 × 10^−7^). This SNP is located near the 5′ end of the 4.1 protein ezrin, radixin, moesin (FERM) domain-containing 3 (*FRMD3*) gene Strong association (p = 3.1 × 10^−6^) also noted in the cysteinyl-tRNA synthase (*CARS*) gene Multivariate Cox proportional hazard analyses showed significant association between both of these genes and time to onset of diabetic nephropathy (*FRMD3:* HR = 1.33, p = 0.02; *CARS*: HR = 1.32, p = 0.01)
19651817	Pezzolesi	2009	GoKinD cohort: 820 DKD, 885 T1DM (284 proteinuria, 536 ESRD)	Caucasian	Affymetrix 5.0 500 K SNP Array 359,193 SNPs (106 genotyped and 12 imputed in ELMO1 locus)	Association of 8 SNPs in *ELMO1* locus with DKD The two strongest associations were found in SNPs located in intron 16 The variant rs7785934 was associated with ESRD No variants were associated with proteinuria
18602983	Bento	2008	300 T2DM-ESRD 310 T2DM	Caucasian	Sequenom MassArray genotyping system 390 SNPs	Identification of 11 significant SNPs/Genes associated with T2DM-ESRD.
15793258	Shimazaki	2005	Discovery: 94 T2DM + DKD, 94 T2DM Replication: 466 T2DM + DKD, 266 T2DM	Japanese	Invader assay 81,315 SNPs	Identification of 1615 loci associated with DKD at a p < 0.01 level *ELMO1* strongly associated with DKD in the replication stage Other 516 polymorphisms in *ELMO1* significantly associated with DKD in a combined analysis of discovery and replication patient and control groups

ACR: Albumin-to-creatinine ratio; AFF3: AF4/FMR2 family member 3; AUH: AU RNA binding methylglutaconyl-CoA hydratase; C2orf48: chromosome 2 open reading frame 48; CARS: cysteinyl-tRNA synthetase; CDH23: cadherin related 23; CI: confidence interval; CKD: chronic kidney disease; CKDGen: Chronic Kidney Disease Genetics Consortium; DCCT/EDIC: Diabetes Control and Complications Trial/Epidemiology of Diabetes Interventions and Complications; DKD: diabetic kidney disease; eGFR: estimated glomerular filtration rate; ELMO1: engulfment and cell motility 1; EPO: erythropoietin; ERBB4: erb-b2 receptor tyrosine kinase 4; ESRD: end-stage renal disease; FinnDiane: Finnish Diabetic Nephropathy Study, GENIE: Genetics of Nephropathy—an international effort Consortium; GALNT11: polypeptide *N*-acetylgalactosaminyltransferase 11; GALNTL5: polypeptide N-acetylgalactosaminyltransferase like 5; GCKR: glucokinase regulator; FRMD3: FERM domain containing 3; GoDARTS: Genetics of Diabetes Audit and Research in Tayside, Scotland; GoKinD: Genetics of Kidneys in Diabetes US Study; GWAMA: genome-wide association meta-analysis; HMGA2: high mobility group AT-hook 2; HPCAL1: hippocalcin like 1; HR: hazard ratio; IL1RAP: interleukin 1 receptor accessory protein; LIMK2: LIM domain kinase 2; MA: meta-analysis; mAlb: microalbuminuria; MCTP2: multiple C2 domains; transmembrane 2; MEOX2: mesenchyme homeobox 2; MSRB3: methionine sulfoxide reductase B3; MYH9: myosin heavy chain 9; NDNN: non-diabetic, non-nephropathy; NPPA: natriuretic peptide A; NPPB: natriuretic peptide B; OR: odds ratio; OSTN: osteocrin; RGMA: RGM domain family, member A; RPS12: ribosomal protein S12; SASH1: SAM and SH3 domain containing 1; SHROOM3: shroom family member 3; SFI1: SFI1 centrin binding protein; SLC7A9: solute carrier family 7 member 9; SNP(s): single nucleotide polymorphism(s); T1DM: type I diabetes mellitus; T2DM: type II diabetes mellitus; UK-ROI: All Ireland Warren 3 Genetics of Kidneys in Diabetes UK Collection; UMOD: uromodulin

While eight *ELMO1* variants showed nominal association with DKD, and variant rs7785934 was found to be significantly associated with the ESRD group, three nominally associated variants (rs1558688, rs741301, and rs7799004) showed opposite genetic effect compared to the previous Japanese cohort study [54]. Ethnic differences may account for differences among studies, as a second, larger study in 1154 patients and 1988 controls of European ancestry from the GENIE consortium also failed to find genome-wide association between DKD and *ELMO1,* but found similar association for other 11 “top-hits” from a previous analysis in European patients [56]. Although performed in an animal model, diabetic mice with increased expression of *ELMO1* show renal dysfunction similar to that seen in human DKD [57]. When the same mutation was induced in both diabetic and non-diabetic mice, *ELMO1* expression was almost twofold higher in the diabetic mice [57]. This may explain why *ELMO1* variants are significantly associated specifically with DKD, but further functional analysis in humans is required.

This study by the GENIE consortium also examined the erythropoietin gene (*EPO*) promoter, previously found associated (variant rs1617640; p = 2.76 × 10^−11^) in a total of 1618 diabetic ESRD patients with proliferative diabetic retinopathy and 954 controls from three independent North American cohorts of European American ancestry [58]. The *EPO* variant rs1617640 was not replicated (although direction of effect was consistent) in the GENIE consortium patients, but the fixed-effects meta-analysis of the original and replication cohorts reached genome-wide significance (p = 2×10^−9^) [56].

### Other traits and kidney diseases

Albuminuria, often measured using UACR, has been previously associated with increased risk of progression to ESRD [59]; therefore, genetic variants associated with increased UACR may help predict CKD progression. A meta-analysis of 63,153 individuals of European ancestry from the CKDGen consortium identified association between variant rs1801239 in the *CUBN* gene and both UACR (*p *= *1.*1 × 10^−11^) and microalbuminuria (*p *= 0.001), defined as UACR > 25 mg/g in women and > 17 mg/g in men [60]. Cubilin, the protein encoded by *CUBN,* when expressed in the kidney, complexes with membrane transporter megalin to reabsorb urinary albumin [61]. Cubilin has been suggested as a biomarker for renal cell carcinoma but may also hold promise as a useful addition to a biomarker panel for CKD [62]. As variants detected often differ among patients of different ethnicities, the rs1801239 variant in the *CUBN* gene was examined in 6981 African American individuals from the CARe consortium, 1159 of which had microalbuminuria. Significant association was again found with both UACR (p = 0.005) and the presence of microalbuminuria, indicating the influence on albuminuria is independent of ethnicity. However larger studies in a wider range of ethnically diverse populations may provide confirmation [60].

As well as showing significant association with eGFR, *SHROOM3* (rs17319721) was also identified as being significantly associated with UACR in a combined meta-analysis of 31,580 and 27,746 Caucasian patients from independent cohorts within the CKDGen Consortium [60]. As this *SHROOM3* variant has also been associated with larger impact on eGFR in patients with albuminuria [4] it may imply that shroom3 has a functional role in proteinuria.

GWAS have also been used to explore other kidney diseases, as shown in Table 4. Association of *HLA*-*DQA1* and *PLA2R1* has been shown in different studies of MGN patients. Both genes were first associated with MGN in three independent cohorts of Caucasian patients versus race-matched controls [63]. Replication of these findings also revealed that while *PLA2R1* only showed association with MGN (p = 1.9 × 10^−8^), but *HLA*-*DQA1* was significantly associated with other renal immune disorders in addition to MGN (p = 5.9 × 10^−27^): LN (p = 2.8 × 10^−6^), T1DM with CKD (*p *= 6.9 × 10^−5^) and FSGS (p = 5.1 × 10^−5^) [64]. As IgAN is more common in Asian individuals [65], many studies have focused on studies of Asian cohorts, to determine if genetic variants give rise to higher disease incidence (Table 4). A combined analysis of 3144 patients and 2822 controls from one European and two Han Chinese cohorts identified five genomic loci significantly associated with IgAN, most of which are involved in innate immune responses [66]. These results were replicated in a meta-analysis of 12 cohorts comprising 5372 cases and 5383 controls of either European, East Asian or African American ethnicity [67]. Twelve SNPs within the five previously reported genomic loci (*CFHR3/R1, HLA DQB1/DRB1, TAP2/PSMB9, DPA1/DPB2, HORMAD2*) were found to reach genome-wide significance (p < 5.0 × 10^−8^) [67]. A study comprising 7658 cases and 12,954 controls of East Asian or European origin replicated all previously reported IgAN-associated variants and identified several new ones [68]. These include new variants within the previously identified *HLA*-*DQ/DR* locus (rs7763262, p = 1.8 × 10^−38^) [66], and the newly identified rs11574637 variant in *ITGAX*-*ITGAM* (p = 2.8 × 10^−1^). Variants within the *HLA* and *ITGAX*-*ITGAM* loci have been identified in patients with other autoimmune disorders, such as rheumatoid arthritis [69] and systemic sclerosis [70], respectively. A study of 8313 Han Chinese cases and 19,680 controls found significant association the *ST6GAL1* (p = 7.27 × 10^−10^), *ACCS* (p = 3.93 × 10^−9^), *ODF1*-*KLF10* (p = 1.41 × 10^−9^) genes, as well as three independent variants in the *DEFA gene* (rs2738058, *p *= 1.15 × 10^−19^; rs12716641, *p *= 9.53 × 10^−9^; rs9314614, *p *= 4.25 × 10^−9^) [71]. The association of a previously reported *ITGAX*-*ITGAM* variant was also validated *(p *= 2.26 × 10^−19^) [68, 71].

**Table 4:** Genomic-wide association studies in other kidney diseases

PMID	Author	Year	Patients	Ethnicity	Methodology	Main findings
27333618	Sekula	2017	Discovery: 323 MGN, 345 non-MGN Sex-replication: Men: 222 MGN/106 non-MGN Women: 101 MGN/239 non-MGN MGN replication: 137 MGN from GCKD, 379 non-MGN	Caucasian	HumanCNV370-Quad SNP chip HumanHap300 SNP chip Discovery: 8.9 million and classical HLA alleles Replication: HLA-DQA1 and PLA2R1	Two previously reported variants in *PLA2R1* and *HLA*-*DQA1* reached genome-wide significance with MGN No additional signals in the pre-replication cohort when separated by men and women Both SNPs were replicated *PLA2R1* was only associated with MGN, but no other CKD aetiology after correction for multiple testing A significant association of the *HLA*-*DQA1* risk variant was observed not only with MGN, but also with lupus nephritis (p = 2.8·10^−6^), CKD in T1DM (p = 6.9·10^−5^) and FSGS (p = 5.1·10^−5^) No association with IgAN
26028593	Li	2015	Combined: 8313 IgAN, 19680 non-IgAN	Han Chinese	GWAMA	Detected novel associations at *ST6GAL1* (rs7634389, p = 7.27 × 10^−10^), *ACCS* (rs2074038, p = 3.93 × 10^−9^) and *ODF1*-*KLF10* (rs2033562, p = 1.41 × 10^−9^) Validated a previously reported association at *ITGAX*-*ITGAM* (rs7190997, p = 2.26 × 10^−19^) Identified multiple independent signals in *DEFA* locus (rs2738058, p = 1.15 × 10^−19^; rs12716641, p = 9.53 × 10^−9^; rs9314614, p = 4.25 × 10^−9^)
25305756	Kiryluk	2014	Discovery: 2747 IgAN, 3952 non-IgAN controls Replication: 4911 IgAN, 9002 non-IgAN	Asian European American	GWAMA Imputation for > 1 million common SNPs	All variants with p-value < 5 × 10^−5^ in the discovery taken forward into the replication analysis Combined meta-analysis of discovery and replication identified 6 novel variants reaching genome-wide significance Four SNPs in 3 novel loci (*VAV3, CARD9* and *ITGAM*-*ITGAX*) were identified, as well as novel a novel SNP in two previously reported regions, *HLA*-*DQ/DR* and *DEFA* Confirmed association of SNPs in *HLA*-*DQ/DR, TAP1/PSMB8, HLA*-*DP, CFHR3, DEFA, TNFSF* and *HORMAD2*
22737082	Kiryluk	2012	8 individual cohorts, combined: 2228 IgAN, 2561 non-IgAN controls	European East Asian African-American	Illumina HumanCNV370-duo Chip	Replication of previous study by Gharavi et al. (2011) Genotyped 12 SNPs in five loci: *CFHR3/R1, TAP2/PSMB9, HLA*-*DPA1/DPB2, HORMAD2* and HLA-*DQB1/DRB1* Ten out of 12 SNPs showed significant association with IgAN, but SNPs in the *TAP2/PSMB9* did not However, all 12 reached genome-wide significance in a meta-analysis of these cohorts and those previously assessed by Ghavari et al. (2011)
21323541	Stanescu	2011	French study: 75 MGN, 157 non-MGN Dutch study: 146 MGN, 1832 non-MGN British study: 335 MGN, 349 non-MGN All racially matched	Caucasian	HumanCNV370-Quad SNP chip French: 315,049 SNPs Dutch: 282,440 SNPs British: 281,009 SNPs	French study: Three SNPs (rs2187668, rs9273327 and rs9272192) in *HLA*-*DQA1* showed significant association with MGN (p = 1.8 × 10^−9^, p = 1.7 × 10^−9^, and p = 5.9 × 10^−10^, respectively) Dutch study: MGN associated with rs2187668 in *HLA*-*DQA1* and a total of 191 SNPs within the extended HLA locus showed significant associations with MGN. Six SNPs within *PLA2R1* also showed association with MGN, all of which reached genome-wide significance (p < 5 × 10^−8^) British study: rs2187668 in *HLA*-*DQA1* showed significant association with MGN (p = 5.2 × 10^−36^) and a further 144 SNPs within the extended HLA locus showed significant association with MGN Two SNPs, rs4664308 and rs187010, showed significant association with *PLA2R1* (p = 2.1 × 10^−10^ and p = 8.2 × 10^−10^, respectively) Combined analysis of all cohorts showed that strongest associations were found in the *HLA*-*DQA1* and *PLA2R1* genes
21399633	Gharavi	2011	Discovery: 1194 IgAN 902 non-IgAN controls Replication: 1950 IgAN, 1920 non-IgAN controls	Discovery: Han Chinese Replication: Chinese and European	Illumina 610 Quad Platform	Twenty-seven SNPs reached genome-wide significance in the discovery analysis, all of which were found within the MHC locus on chromosome 6 Sixty-seven SNPs in 10 other distinct loci showed nominal significance (p < 1.3 × 10^−5^) and the top 20 with the lowest p-values were taken forward for follow-up Five SNPs reached genome-wide significance in the replication analysis: The strongest variant showing strongest association with IgAN was rs9275596 (p = 1.6 × 10^−26^), which is found in a region containing the *HLA*-*DRB1*/*DQA1*/*DQB1* locus Variant rs9357155 was significantly associated with region containing *TAP2, TAP1, PSMB8* and *PSMB9* genes (p = 6.9 × 10^−9^) Genome-wide significance was also reached for variant rs9275596 in the *HLA*-*DPA1/DPB1/DPB2* gene region (p = 3.1 × 10^−8^) Variant rs6677604 in the *CFH* region containing the *CHFR1, CHFR2, CHFR3, CHFR4* and *CHFR5* genes showed significance association with IgAN (p = 3.0 × 10^−10^) The final variant reaching genome-wide significance was rs2412971 in the *HORMAD2* gene

ACCS: 1-aminocyclopropane-1-carboxylate synthase homolog (inactive); CARD9: caspase recruitment domain family member 9; CFH: complement factor H; CFHR1: complement factor H related 1; CFHR2: complement factor H related 2; CFHR3: complement factor H related 3; CFHR4: complement factor H related 4; CFHR5: complement factor H related 5; CKD: chronic kidney disease; DEFA: defensins; alpha; FSGS: Focal segmental glomerulosclerosis; GCKD: German Chronic Kidney Disease; GWAMA: genome-wide association meta-analysis; HLA-DP: major histocompatibility complex, class II, DP; HLA-DPA1: major histocompatibility complex, class II, DP alpha 1; HLA-DPB1: major histocompatibility complex, class II, DP beta 1; HLA-DPB2: major histocompatibility complex, class II, DP beta 2; HLA-DQA: major histocompatibility complex, class II, DQ alpha; HLA-DQA1: major histocompatibility complex, class II, DQ alpha 1; HLA-DQB1: major histocompatibility complex, class II, DQ beta 1; HLA-DQR: major histocompatibility complex, class II, DR; HLA-DRB1: major histocompatibility complex, class II, DR beta 1; HORMAD2: HORMA domain containing 2; IgAN: immunoglobulin A nephropathy; ITGAM: integrin subunit alpha M; ITGAX: integrin subunit alpha X; KLF10: Kruppel like factor 10; MA: meta-analysis; MGN: membranous glomerulonephritis; MHC: major histocompatibility complex; ODF1: outer dense fiber of sperm tails 1; PLA2R1: phospholipase A2 receptor 1; PSMB8: proteasome subunit beta 8; PSMB9: proteasome subunit beta 9; SNP: Single nucleotide polymorphism; ST6GAL1: ST6 beta-galactoside alpha-2,6-sialyltransferase 1; T1DM: type I diabetes mellitus; TAP1: transporter 1; ATP binding cassette subfamily B member; TAP2: transporter 2; ATP binding cassette subfamily B member; TNFSF: tumour necrosis factor superfamily; VAV3: vav guanine nucleotide exchange factor 3

### Conclusions

Although several genes with strong associations to renal diseases, eGFR and UACR, have been identified, the development of a universal diagnostic gene biomarker panel is however challenged by the variability and ethnic specificity of many of the variants identified. While GWAS may provide insights to genetic causes of disease, little information regarding the functional implications of the identified SNPs can be derived. Further analyses such as epigenomic and transcriptomic profiling, coupled with protein and metabolite analysis may give a more complete picture of CKD. A multi-omic approach may add functional and physiological evidence for the use of GWAS-identified variants as biomarkers or lead to the identification of biomarkers of a different nature that have not been detected through GWAS.

## Epigenetic biomarkers

Epigenetics is defined as, “the study of changes in gene function that are mitotically and/or meiotically heritable and that do not entail a change in DNA sequence” [72]. Epigenetics acts as a bridge between genotype and phenotype, helping to explain why some genetic alterations may not necessarily result in an altered phenotype. It is therefore extremely important to consider epigenomic biomarkers alongside genomic and transcriptomic analyses to discover the role of different pathways in disease, as change in gene expression may not be linked solely to genetic aberrations and therefore will not be detected using purely genetic analysis.

Epigenetics can be subdivided by mechanism. Covalent modifications of DNA and histone proteins, such as DNA methylation, and the action of non-coding RNAs, such as microRNAs (miRNAs), all seek to interfere with gene expression without changing the genetic sequence of the gene in question, including any of its promotor or enhancer regions. Epigenetic regulation of gene expression results in altered levels of target gene mRNA available for translation, and thus affects protein synthesis. Epigenetic regulation is required to maintain the regulation of gene activity, transcription and contributes to the maintenance of genomic stability [73]. However, epigenetic dysregulation has previously been linked to diseases like Prader–Willi syndrome, an imprinting disorder, Fragile X syndrome, caused by loss of genomic stability and multiple different cancers [74]. Evidence has suggested that loss of epigenetic control may be associated with CKD, with differential expression of microRNAs [75] and differential DNA methylation detected in kidney fibrogenesis, a hallmark of CKD [76].

### DNA methylation

DNA methylation is an example of a covalent modification to the DNA sequence that has the ability to alter gene transcription, especially if found near the promoter or enhancer regions of genes [77, 78]. DNA methylation is the transfer of a methyl group (-CH_3_) from *S*-adenosylmethionine to the fifth carbon of a cytosine nucleotide in the DNA sequence [79], catalysed by DNA methyltransferases (DNMTs) [80, 81]. It is the presence of these cytosine-bound methyl groups that can either prevent the binding of transcription factors in the promoter region of genes, or attract and bind repressor proteins, both of which lead to a reduction in gene transcription. The majority of methylated cytosines are followed by a guanine nucleotide and these sites have been denoted as CpG sites [82]. Most of CpG sites are methylated, except in regions of high CpG density, known as CpG islands. CpG islands exhibit much lower levels of DNA methylation than expected, with methylation found to be significantly lower in these regions when compared to other genomic CpG sites [83]. CpG islands are often found at gene promoters, and their methylation regulates gene expression [84]. DNA methylation has been shown to be involved in maintaining X-inactivation [85], the silencing of imprinted genes [86], and plays a role in embryonic development; knockout of DNMT enzymes in mice results in embryonic lethality [87, 88]. The role of DNA methylation in diseases like cancer is clear, with colorectal, breast and renal cancers all showing high levels of epigenetic dysregulation [89].

#### Role of DNA methylation in CKD

Multiple approaches have been taken to further understand the role of epigenetic aberrations in the pathogenesis of CKD (Table 5). Global methylation profiling [90–93], epigenome-wide association studies [94–101] and candidate gene studies [91, 102, 103] have been carried out in patients with CKD, DKD and ESRD. Detection of differential DNA methylation in these studies may lead to new diagnostic and prognostic epigenetic biomarker panels being developed.

**Table 5:** Methylation studies in chronic kidney disease

PMID	Author	Year	Sample	Patients (n)	Controls (n)	Ethnicity	Methodology	Main findings
28609449	Bailie	2017	Blood	CKD (n = 255)	No evidence of CKD (n = 152)	Caucasian	450 K array 485,577 CpG sites: *UMOD1*, *UMOL1* and *UMODL1*-*AS1* genes	Differential methylation of three CpG sites in *UMOD*. The most significant was cg03140788 (p = 3.7∙10^−10^) Eight associated CpG sites in *UMODL1* and *UMODL1*-*AS1* genes, with genome wide significance. The most significant for non-diabetic ESRD was cg16624482 (p = 2.9∙10^−32^)
28755807	Zinellu	2017	Blood	CKD (n = 30)	Healthy (n = 30) Age- and sex-matched controls	Italy	Percentage of methylated to total cytosine	Lower DNA methylation in CKD (4.06 ± 0.20% vs. 4.27 ± 0.17%; p = 0.0001 Cholesterol lowering treatment significantly increased DNA methylcytosine concentrations in all patients (4.06 ± 0.04% at baseline; 4.12 ± 0.03% at 4 months; 4.17 ± 0.03% at 8 months; and 4.20 ± 0.02% at 12 months; p = 0.0001 for trend) The combined 10/40 mg/day ezetimibe/simvastatin treatment showed a trend for a greater effect on DNA methylation (+5.2% after one year treatment)
27018948	Zawada	2016	Differentiated intermediate monocytes from haematopoietic stem cells, isolated from healthy donors	ESRD (n = 5) RRT	Healthy (n = 5) Age- and sex-matched controls	Germany Primary culture (Treatment with serum from CKD patients (uraemic) or control patients)	Methyl-Seq 813,267 Genomic loci	Patients had higher counts of intermediate (p < 0.05) and nonclassical (p < 0.01) monocytes Upon stimulation with uremic serum, more intermediate monocytes were generated as compared to control conditions (p < 0.001) Monocytes generated under uremic conditions were closer related to circulating intermediate monocytes than monocytes generated under control conditions Five of the top 50 DMRs included in the comparison between “CKD” and “control” monocytes had been found involved with CKD by other authors
24363223	Ghattas	2014	Blood	ESRD (n = 96)	Healthy (n = 96)	Egyptian	Methylation-specific PCR *MTHFR* gene	The frequency of *MTHFR* methylation was significantly higher in ESRD patients (p = 0.003) eGFR was significantly lower when *MTHFR* promoter was methylated (p = 0.026)
24253112	Smyth	2014	Blood	CKD (n = 255)	No evidence of CKD (n = 152)	Caucasian	450 K array 485,577 CpG sites	More than one significantly affected CpG in 23 unique genes after adjustment (p < 10^−8^) RNA-seq data also supported a functional role for DM in *ELMO1* and *PRKAG2*, as they saw altered expression in the expected manner, even if change in *PRKAG2* expression was not statistically significant *CUX1*, *ELMO1*, *FKBP5*, *INHBA*-*AS1*, *PTPRN2*, and *PRKAG2* genes proposed as biological candidates for kidney disease
24516231	Wing	2014	Blood	“Rapid Progression” CKD (n = 20) eGFR 20–70 ml/min/1.73 m^2^ “Highest Rate of Decline” CRIC cohort	“Stable Kidney Function” CKD (n = 20) eGFR 20–70 ml/min/1.73 m^2^ “Slowest Rate of Decline”	Caucasian (50%) African American/Black (40%) Oher (10%)	450 K array 485,577 CpG sites	No gene reached significance after FDR Of the 15 genes most strongly associated with rapid progression, 14 of them were more heavily affected in the slow progression group *UMODL1* among several others, proposed as “top candidates”
24224012	Chen	2013	Renal biopsy/PBMC	CKD (n = 47)	Renal cell carcinoma (Renal biopsy, n = 47) Healthy (PBMC, n = 46)	China	Pyrosequencing: 6 CpGs assessed in Klotho (*KL*) Gene	*KL* gene was hypermethylated in 68% tissue and 70% PMBC samples from CKD patients when compared to controls, significance reached across all CpG sites (p < 0.001) Methylation of PBMC *KL* positively correlated with renal KL methylation (p < 0.001) eGFR inverse correlation with renal and PBMC levels of *KL* promoter methylation (both p < 0.001)
18287179	Nanayakkara	2008	Genomic DNA extracted from Leukocytes	CKD 2–4 (n = 78) (eGFR 15-70 ml/min/1.73 m^2^) ATIC study	109 healthy volunteers	The Netherlands	LC–MS	No association was found between DNA methylation and renal function
18067454	Geisel	2007	PBMC	ESRD (n = 22) all receiving RRT (22 HT, 20 DM and 14 CVD)	Healthy (n = 26). Age- and sex-matched	Germany	Pyrosequencing: LINE-1 (nCpG = 7) p66Shc Promoter (nCpG = 3)	Methylation of p66Shc promoter significantly reduced in ESRD patients (p < 0.01). LINE-1 (used as surrogate marker of global methylation) significantly hypermethylated in ESRD patients (p < 0.01)
17444888	Stenvinkel	2007	Peripheral blood leukocytes	4 Groups: Group 1: Non-Inflamed (CRP < 10mgL-1) stage 3 or 4 (7 GN, 1 DKD, 4 PCKD, 5 = nephrosclerosis, 5 = other, 6 = unknown) Group 2A: stage 5 + RRT + Non-inflamed (n = 56) Group 2B: stage 5 + RRT + Inflamed (n = 42). Group 3: stage 5 + RRT + Constant Inflammation (weekly CRP measurements for 3 months) (n = 20)	Group 4: Healthy controls randomly selected from the population (n = 36)	Sweden	LUMA HpaII/MspI ratio	No association found between DNA methylation and eGFR No differences found between diabetic and non-diabetic patients Association of global hypermethylation with CVD (p < 0.01) Inflammation associated with global hypermethylation (p = 0.0001) In essence, the association was with inflammation, not CKD

ATIC: Anti-Oxidant Therapy In Chronic Renal Insufficiency study; CKD: chronic kidney disease; CpGs: CpG islands or CG islands (5′—C—phosphate—G—3′: cytosine and guanine separated by only one phosphate group); CRP: C-reactive protein; CRIC: Chronic Renal Insufficiency Cohort; CUX1: cut like homeobox 1; CVD: cardiovascular disease; DM: diabetes mellitus; DKD: diabetic kidney disease; DMR: differentially methylated regions; DNA: deoxyribonucleic acid; eGFR: estimated glomerular filtration rate; ELMO1:engulfment and cell motility 1; ESRD: end-stage renal disease; FDR: false discovery rate; FKBP5: FK506 binding protein 5; HT: hypertension; INHBA-AS1: INHBA antisense RNA 1; KL: klotho; LC–MS: liquid chromatography-tandem mass spectrometry; LINE-1: Long interspersed nuclear element 1; LUMA: luminometric methylation assay; MTHFR: methylenetetrahydrofolate reductase; p66Shc: p66 isoform of the SHC-transforming protein 1; PBMC: peripheral blood mononuclear cells; PCKD: polycystic kidney disease; PRKAG2: protein kinase AMP-activated non-catalytic subunit gamma 2; PTPRN2: protein tyrosine phosphatase, receptor type N2; RRT: renal replacement therapy; UMOD: uromodulin; UMODL1: uromodulin like 1; UMODL1-AS1: UMODL1 antisense RNA 1

##### Global methylation and CKD

The influence of total genomic methylation levels on CKD has been analysed by global methylation analysis. Global methylation can be assessed by different methodologies; enzymatic digestion with methylated and unmethylated cytosine-sensitive enzymes generates a methylation ratio using the luminometric methylation assay (LUMA), percentage methylation can be established using a combination of liquid chromatography and mass spectrometry (LC-MS) or surrogate markers of whole genome methylation may be used [104]. An example of the latter is pyrosequencing the long-interspersed nuclear element 1 (LINE-1), which is considered a reliable marker of global methylation [104, 105].

In the first study of global methylation in CKD, LUMA was used to evaluate 155 patients with CKD (stage 3–5) and 36 healthy controls [90]. A differential methylation ratio was found for inflammation in late-stage CKD patients CKD (*p *= 0.0001) [90]. While this study showed no significant difference for CKD itself, other studies attempted to demonstrate differential global methylation in CKD, with mixed success. Pyrosequencing of the LINE-1 element in 22 ESRD patients and 26 healthy controls showed that this retrotransposon was significantly hypermethylated (*p *< 0.01) in ESRD patients [91]. Although all ESRD patients also had hypertension, this is not known to be associated with global hypermethylation, being either associated with global loss of methylation [106] or with no difference in methylation at the LINE-1 transposable element [107]. While this study found significant hypermethylation associated with ESRD, another study focusing on the association between DNA methylation and renal function in 93 CKD patients (stage 2–4) found no significant association of DNA methylation with renal function [93]. Indeed, a more-recent study found significant DNA hypomethylation (*p *= 0.0001) in 30 Stage 3–4 CKD patients and demonstrated the ability of cholesterol lowering agents to significantly increase DNA methylation [92].

No clear association of CKD with global changes in DNA methylation has been established from these studies. Published studies of global methylation have reported on patient groups with small numbers (n < 100) and such studies lack power to detect significant differences in global DNA methylation.

##### Site-specific differential methylation in chronic kidney disease

In the last 10 years, research has been undertaken to identify individual genes or genomic regions whose differential methylation is associated with CKD; several groups have utilised large, array-based methods to analyse DNA methylation at an epigenome-wide level. The Illumina Infinium HumanMethylation 450 K BeadChip (450 K Array) is used to assess methylation status of 485,577 genomic CpG sites [108], while next-generation methylation sequencing (Methyl-Seq) assesses the methylation status of 813,267 CpG sites [109]. These large, array-based methods allow much higher sample throughput, contrasting with traditional techniques for assessing methylation like pyrosequencing, where only a fraction of the CpG sites can be assessed in the same time-frame.

Using the 450 K array, 23 unique genes were identified in a sample of 255 CKD patients that contained more than one significantly differentially methylated CpG site (*p *< 10^−8^) [97]. Of these genes, six were selected to undergo RNA analysis. While some genes, such as *ELMO1*, showed altered levels of expression in CKD patients, these differences did not reach significance. So while this may suggest that DNA methylation plays a role in regulating their expression, further analysis is required in a larger number of patients, as only two Stage 5 CKD patients and two controls were used in expression studies [97]. Further study in this group of patients was focused on the differential methylation of the *UMOD* and related genes *UMODL1* and *UMODL1*-*AS1* genes [100], previously associated with CKD in GWAS [23, 25]. Indeed, 11 CpGs across the three genes were found to be significantly differentially methylated in patients with CKD (*p *< 10^−8^) [100].

The relationship between DNA methylation and CKD progression has also been assessed in Stage 2–4 CKD patients. Twenty patients with “rapid disease progression” and 20 patients with “stable kidney function,” determined using a mixed effects linear regression model using two measures of eGFR, had their methylation status assessed using the 450 K Array [98]. Although no significant association with CKD progression could be found after correction for multiple testing, several candidate genes (including *UMODL1*) that had previously been related with CKD in the literature [96, 98, 100] showed a trend towards association with rapid CKD progression.

Differential methylation of single genes has also been studied, particularly when they are candidate to have a functional role in the development of CKD. In a study of 96 ESRD patients/96 controls, the specific methylation of the methylenetetrahydrofolate reductase (*MTHFR*) gene promoter was found to be significantly associated with ESRD (p = 0.003) and significant correlation was observed between a lower eGFR and *MTHFR* promoter methylation (p = 0.026) [102]. The hypermethylation of the *MTHFR* promoter is thought to lead to the transcriptional silencing of *MTHFR*, preventing remethylation of *S*-adenosylhomocysteine to S-adenosylmethionine, which is required to donate methyl groups necessary in DNA methylation [110]. Loss of MTHFR activity is characterised by a build-up of *S*-adenosylhomocysteine, resulting in hyperhomocysteinaemia in patients—which is strongly associated with CKD [111]. However, *MTHFR* was also highlighted in two other analyses, with Sapienza et al. and Ko et al. both finding significant hypomethylation of the *MTHFR* gene in patients with ESRD and DKD and solely DKD, respectively [95, 96]. This contrasts with the hypermethylation of the gene promoter found in the original study. Therefore, as the evidence of *MTHFR* as a biomarker for CKD remains conflicting, its usefulness in any panel of non-invasive epigenetic biomarkers remains controversial [95, 96, 102].

Significant hypomethylation at the promoter of *SHC1*, formerly known as *P66SHC* (Gene ID: 6464), was found in 22 patients with ESRD (p < 0.01) [91]. *SCH1* is involved in the regulation of reactive oxygen species [112] and may be linked to CKD due to oxidative stress has been shown increased in patients with CKD [113].

Klotho (*KL*) is another gene that has been assessed on an individual basis in CKD patients. *KL* is highly expressed in kidney tissues, and causes a syndrome similar to CKD when it is knocked out in mice [114]. Both *KL* mRNA and protein production have been found to be reduced significantly in patients suffering from ESRD [115]. Using pyrosequencing, six CpG sites across the *KL* gene in peripheral blood DNA and DNA extracted from renal tissue of 47 patients were analysed [103]. Forty-seven renal cell carcinoma biopsies and peripheral blood DNA of 48 healthy patients acted as non-CKD control samples. *KL* was significantly hypermethylated in approximately 70% of all renal and blood samples taken from patients (p < 0.001), and *KL* promoter hypermethylation also found to be inversely correlated with eGFR [103]. For this reason, *KL* has been further investigated in animal and in vitro models to determine if KL promoter hypermethylation is involved in the development of CKD and altered renal fibrosis was noted in cells with *KL* hypermethylation [116, 117].

#### Association with diabetic kidney disease

To date, several studies have investigated the role of methylation in DKD and diabetic ESRD, as shown in Table 6 [94–96, 101].

**Table 6:** Methylation studies in diabetic kidney disease

PMID	Author	Year	Sample	Cases (n)	Controls (n)	Ethnicity	Methodology	Main findings
25850930	Swan	2015	Blood	DKD (n = 100) DKD + ESRD (n = 50) RRT	Diabetes and no renal disease (n = 100)	White	27 K 450 K Mitochondrial Genes	Identification of 450 CpGs differentially methylated T1DM-DKD: 54 probes (51 genes) significant (p < 10^−8^) *PMPCB*, *AUH* and *TSFM*: DMRs at more than one CpG ESRD: identification of 755 CpG probes in 374 (p ≤ 10^−8^). Forty-three top-ranked CpG sites for DKD were also differentially methylated identified in the subgroup of patients with ESRD
24098934	Ko	2013	Tubule epithelial cells	Discovery: CKD (both HT and DKD) (n = 14) Replication: DKD (n = 21)	Discovery: Healthy (n = 14) Replication: Non-DKD	Mixed	Discovery: MSP-HELP Replication: MassArray Epityper (INTERNAL) and 450 K (external)	Identification of 4751 DMRs identified in Discovery, 1535 unique genes, 70% showed hypomethylation in CKD Confirmation of 1061 unique genes were identified in the replication cohort (98% of genes found in the discovery analysis) *COLIVA1*, along with 5 other gene loci, had mRNA/protein analyses performed DMRs enriched in kidney-specific gene regulatory regions (mainly enhancers/binding motifs for renal-specific transcription factors
21150313	Sapienza	2011	Saliva	DM-ESRD (n = 23)	DM (n = 23)	African American Hispanic American	27 K array 27,578 CpG sites	Due to the small number of patients involved, only genes which had at least two significant CpGs were considered as “candidate” biomarker genes. This yielded 187 genes (389 CpGs) When Benjamini–Hochberg correction applied, original no. of associated CpGs fell from 2870 (approximately 10%) to 30
20687937	Bell	2010	Blood	DKD (n = 96)	T1DM	Irish	27 K array 27,578 CpG sites	Over 400 differentially methylated CpG sites found (263 conferred increased risk and 162 conferred decreased risk for DKD) Narrowed down to 19 CpGs when more stringent FDR threshold (0.05) applied Many genes overlap with Ko et al. (2013) at less stringent threshold, but only one (*RUNX3)* when FDR threshold reduced to 0.05

AUH: AU RNA binding methylglutaconyl-CoA hydratase; CKD: chronic kidney disease; CpGs: CpG islands or CG islands (5′—C—phosphate—G—3′: cytosine and guanine separated by only one phosphate group); DKD: diabetic kidney disease; DM: diabetes mellitus; DMRs: differentially methylated regions; MSP-HELP: methylation-sensitive and -insensitive isoschizomer enzymes (HpaII and MspI) followed by (HpaII) fragment enrichment by ligation-mediated PCR; HT: hypertension; RRT: renal replacement therapy; T1DM: type 1 diabetes mellitus; PMPCB: peptidase, mitochondrial processing beta subunit; RUNX3: runt related transcription factor 3; TSFM: Ts translation elongation factor; mitochondrial

Two different studies have used the Illumina Infinium HumanMethylation27 BeadChip (27 K Array) to assess the methylation status of 27,578 CpG sites in patients with DKD and diabetic ESRD [94, 95]. Bell et al. examined differences in DNA methylation in genomic DNA extracted from whole blood from 96 DKD patients and 96 controls [94], while Sapienza et al. focussed on differential methylation in DNA extracted from saliva of 23 diabetic ESRD patients and 23 controls [95]. Bell et al. found over 400 differentially methylated CpGs originally, which were reduced to 19 when corrected for multiple testing. Sapienza et al. found 187 genes containing more than one significantly differentially methylated CpG, but several of these genes were eliminated as false-positives after correction. There were no common genes identified between these two groups. Although they used the same methylation analysis, this is perhaps not surprising, since Sapienza studied patients with diabetic ESRD, whereas Bell only analysed patients with DKD, and both studies were very small. Ethnic differences in cohorts may also have played a role; Bell et al. analysed samples from Irish patients [94], where Sapienza examined African American and Hispanic American patients [95]. Indeed, when Hispanic patients were removed from the analysis, the number of genes detected as being differentially methylated was reduced, showing that ethnicity may influence on the genes detected [95]. While these studies have not provided clear differentially methylated candidate genes, they have highlighted the need for studies with greater statistical power and that the future of epigenome-wide studies may lie in the analysis of specific phenotypes within well-defined patient populations.

By far the most extensive list of differentially methylated genes in DKD has been produced by Ko et al. where 14 patients were compared to 14 controls in a discovery analysis, which was followed up by a replication study consisting of 21 patients and 66 controls [96]. There were marked differences in phenotypes between the discovery and replication patient groups; the discovery cohort had both hypertension and DKD patients, and controls were healthy subjects, whereas the replication cohort consisted of a DKD patient group and non-DKD control group, including patients with hypertension and diabetes. They identified 1061 unique differentially methylated genes in the replication cohort, 98% of which had also been found in the discovery analysis. Of the 2% of genes only found in the discovery cohort, *CUX1*, *PTPRN2* and *STK24* had previously been associated with CKD [97] and the association of *SALL1* and *WHSC1* were later confirmed in cells extracted from non-diabetic ESRD patients [99]. These genes were not found to be associated in the replication analysis, suggesting that even subtle phenotypic differences may give rise to differences in DNA methylation. Further analysis would be required to determine the contribution of specific phenotypes to the significance associated with CpGs in these genes. The larger number of significant differentially methylated CpGs detected in this study may also be due to the use of renal tubule cells as opposed to genomic DNA from whole blood extracted in all previous epigenome-wide analyses.

*RUNX3* and *PHB* have been described in multiple studies analysing differential methylation in DKD. *RUNX3* was found to be significantly differentially methylated by Ko et al. and Bell et al. and in fact was the only unique gene that remained significant after the application of a more stringent false discovery rate threshold [94, 96]. RUNX3 is a transcription factor that works in balance with the STAT5 transcription factor in the renal fibrosis pathway [118], suggesting that altered methylation and thus expression of *RUNX3* may lead to aberrant renal fibrosis, a hallmark of CKD [119]. *PHB*, a mitochondrial gene, was identified by both Ko et al. and Swan et al. Swan et al. found *PHB* significantly differentially methylated in 150 DKD patients, 50 of whom were diabetic ESRD patients, and significance was reached for *PHB* in both groups when compared to diabetic controls [101]. The sub-analysis in mitochondrial genes showed 51 genes differentially methylated in DKD versus the diabetic control group (*p *< 10^−8^), and 374 genes were identified between the ESRD and diabetic control group [101]. Forty-three of these genes overlapped with the 51 genes found associated with DKD, but *PHB* was the only gene overlapping with other studies [96, 101]. *PHB* has been involved in regulating cellular processes, such as transcription and apoptosis [120] and its dysregulation increases renal fibrosis and oxidative stress [121], which have previously been associated with CKD [113, 119].

In a non-population based study, further analysis of the epigenetic changes involved in ESRD was investigated. The genome-wide methylation of monocytes treated with either serum from patients with ESRD or healthy controls was assessed [99]. Although only performed in 5 patients and 5 healthy age- and sex-matched controls, differentially methylated CpGs were detected between monocytes that had differentiated in the presence of ESRD patient or control serum. The top 50 genes were listed and five of these (*GPR39*, *HDAC1*, *PRKCE*, *SLC43A2* and *ST3GAL5*) overlapped with genes identified as significant in the replication analysis by Ko et al. [96, 99]. While this study seeks to replicate the changes in DNA methylation seen in uraemic ESRD patients rather than measuring DNA methylation in patients directly, overlapping results in DKD patients may prompt further investigation in this area in a large patient cohort to confirm results at population level.

Additional work has been carried out by several groups to determine the functional implications of the significant differences in methylation in CKD patients [96, 97]. Ko et al. performed RNA and Gene Ontology analysis on the replication cohort and found that over 40% of the significantly methylated genes showed significant changes in gene expression. Many of these genes, such as *TGFBR3* and *SMAD6,* belong to the TGF-β pathway, whose involvement in CKD development is well-established [96]. Smyth et al. carried out similar analysis on two patients and controls and although no significant changes in gene expression were found, pathway analysis linked several genes to the mucin type O-glycan biosynthesis pathway, dysregulation of which has been shown to result in kidney abnormalities [97, 122]. However, despite this additional work, no genes or CpG sites have been established as a clear candidates to be taken forward into biomarker development [96, 97].

### Conclusions

Altogether, these findings suggest a role for DNA methylation analysis in the diagnosis and prognosis of CKD and related diseases, but also highlight the difficulties associated with establishment of definitive epigenomic biomarkers. Among all the methylation markers described in the literature, only a few genes appear more than once. *MTHFR* gene is the only epigenetic biomarker proposed in more than two studies, but with inconsistent results regarding the mechanism underlying its influence [95, 96, 102]. Therefore, the role of *MTHFR* as a potential biomarker in CKD is yet to be elucidated. Further investigations using a larger number of CpG sites, phenotypes and ethnicities in larger cohorts is required to allow accurate, phenotype-specific epigenetic biomarkers to be established. Deeper understanding of the role DNA methylation plays in disease development may lead to the discovery of potential points of therapeutic intervention, as well as the discovery of diagnostic and prognostic biomarkers. To better understand this, the identification of specific methylation regions in CKD is essential, to serve both as potential candidates for epigenomic biomarkers, but to also to give an indication of the genes affected in different functional pathways, giving rise to the elucidation of mechanisms involved in the development of CKD.

### microRNA

MiRNAs are 21–25 nucleotides length small non-coding RNA molecules, partially complementary to one or more messenger RNA (mRNA), which downregulate gene expression by translational repression, mRNA cleavage, and deadenylation. Targeted mRNAs include those involved in biological processes such as inflammatory response, cell–cell interaction, apoptosis and intra-cellular signalling [123]. MiRNAs have been widely investigated as diagnostic and prognostic biomarkers in CKD, with a particular interest in their potential use as non-invasive markers.

#### Chronic kidney disease

##### Prediction of CKD

Several studies have explored the use panels of differentially expressed miRNAs as potential biomarkers for CKD although these reported on relatively small numbers of patients (Table 7).

**Table 7:** miRNA studies in chronic kidney disease

PMID	Author	Year	Sample	Patients	Ethnics	Methodology	Main findings
27872161	Khurana	2017	Urine exosomes	15 CKD	Caucasian	Ion proton system for NGS ncRNASeqScan	Sixteen miRNAs differentially expressed Increased: let-7c-5p, miR-222-3p, miR-27a-3p, miR-27b-3p, miR-296-5p, miR-31-5p, miR-3687, miR-6769b-5p, and miR-877-3p Decreased: miR-133a, miR-133b, miR-15a-5p, miR-181a-5p, miR-34a-5p, miR-181c-5p, and miR1-2
28077372	Muralidharan	2017	28 CKD		Affymetrix GeneChip miR 4.0		
			Urine Blood (circulating)				Differential expression of 384 urinary and 266 circulatory miRNAs between CKD patients with eGFR≥30 vs. < 30 ml/min/1.73 m^2^ Pathway analysis mapped multiple miRNAs to TGF-β signalling-related mRNA targets Urine downregulation of let-7a and upregulation of miR-130a in patients with eGFR < 30 ml/min/1.73 m^2^ Both urine and plasma upregulation of miR-1825 and miR-1281 in patients with decreased eGFR Plasma downregulation of miR-423 in patients with decreased eGFR
28771472	Nandakumar	2017	Blood	15 CKD 15 non CKD	African American	RNA sequencing (347 miRNAs)	Fourteen significant miRNAs: Upregulated: miR-29c-5p, miR-345-5p, miR-142-3p, miR-339-3p Downregulated: miR-17-5p, miR-130a-3p, miR-15b-5p, miR-106b-3p, miR-106a-5p, miR-16-5p, miR-181a-5p, miR-1285-3p, miR-15a-5p, miR-210-3p The most significant miRNA was miR-17-5p (log2 fold change = − 0.77; p = 6.7∙10^−4^) Downregulation of two other miRNAs in the miR-17 family (miR-106a-5p, miR-106b-3p) Downregulation of three members of miR-15 family (miR-15a-5p, miR-15b-5p, and miR-16-5p)
27650800	Rivoli	2017	Blood (extra-cellular vesicles)	30 CKD 17 ESRD pre-HD 15 post-kidney transplantation 22 HC		qRT-PCR: miR-122 miR-885	Total circulating miRNA was lower in patients with CKD miR-122 not differentially expressed in CKD Downregulation of circulating miR-122 in pre-HD (19-fold lower compared with healthy controls miR-122 not differentially expressed in patients with renal transplantation Downregulation of miR-885 in ESRD patients (fourfold compared to healthy subjects) and upregulation by haemodialysis and renal trans-plantation No correlation between eGFR and miR-122 in 30 patients with CKD Circulating miR-122 was substantially reduced in ESRD patients pre-HD compared with the other groups (19.0-fold lower than healthy controls; 21.7-fold lower than CKD) Downregulation of miR-885 in ESRD patients (fourfold compared to healthy subjects) and was increased by haemodialysis Haemodialysis increased the concentration of miR-122
26267685	Zhang	2015	Serum	82 CKD-uremic, 16 HC		miR-155	Serum upregulation of miRNA-155 and IL-6 in all dialysis patients (p < 0.05) miRNA-155 expression in peritoneal dialysis treated with alprostadil was down-regulated compared with peritoneal dialysis group or HD group (p < 0.05 Serum miRNA-155 was positively correlated with the level of IL-6 as well as CRP, while miR-155 was negatively correlated with HDL and albumin. Alprostadil could ameliorate the inflammatory conditions of uremic dialysis patients by inhibition of the IL-6 expression. Serum miRNA-155 may be a novel target for the treatment of uremic dialysis patients
25197634	Hu Yang-Yang	2014	Serum	60 Nephrolithiasis, 50 HC	Asian	qRT-PCR: miR-155	Overexpression of serum and urinary levels of miR-155 in nephrolithiasis patients eGFR inversely correlated with urinary level of miR-155
24184689	Zawada	2014	PBMC	10 HD 10 HC		Massive analysis of cDNA ends	One-hundred and eighty-two miRNAs differentially expressed (p < 0.001) between HD patients and control subjects (75 upregulated in HD patients, 107 downregulated Among the differentially expressed miRNAs, miR-21, miR- 26b, miR-146b, or miR-155 are involved in cardiovascular disease
23717629	Chen	2013	Blood (Circulating)	90 CKD	(Caucasians: African Americans: Others) 51:38:1	qRT-PCR: miR-125b miR-145 miR-155	Circulating levels correlated with progressive loss of eGFR by multivariate analyses. (For every one ml/min decrease in eGFR, there were a 0.05, 0.04, and 0.02 unit decreases in the expression of the miRNA for miR-125b, 145, and 155, respectively
				10 CKD 3–4 10 HD 8 HC			Downregulation of circulating levels of miR-125b, miR-145 and miR-155 in CKD and hemodialysis patients compared to healthy volunteers
23946286	Lv	2013	Urine Exosomes	32 CKD 7 HC	Asian	qRT-PCR: miR-29a miR-29b miR-29c miR-200a miR-200b miR-200c	Downregulation of all 6 miRNAs in CKD Downregulation of members of miR-29 and miR-200 families (p < 0.05) in urine, being able to distinguish CKD from controls miR-29c correlated with both eGFR (r = 0.362; p < 0.05) and degree of tubulointerstitial fibrosis (r = -0.359; p < 0.05) for CKD patients miR-29a and miR-29c could predict degree of tubulointerstitial fibrosis
22836280	Plé	2012	Platelets	10 CKD 5 HC		mRNA profiling	The levels of most miRNAs appeared to be corrected by dialysis, showing the regulatory role of platelet microRNAs in dialysis Upregulation of miR-19b, a miRNA involved in platelet reactivity miR-551b was found to be differentially expressed in the dialysis patients cohort, as compared to the healthy cohort
21891774	Neal	2011	Plasma (Circulating)	15 CKD-3 18 CKD-4 20 ESRD-HD 22 HC		Total level of small RNAs (18–25 nucleotides)	Downregulation of specific circulating miRNAs in patients with severe chronic renal failure, compared to patients with mild renal impairment or normal renal function Correlation between circulating miRNAs and eGFR (p < 0.0001; r = 0.553)
							Total small RNA concentration in plasma differentially expressed between normal/stage 3 and stage 4/ESRD (p < 0.0001; Mann–Whitney U-test)
						qRT-PCR: miR-16 miR-21 miR-155 miR-210 miR-638	All five miRNAs expression inversely correlated with kidney function (eGFR) Significant differences were observed between normal and Stage 4 CKD for miR-210, miR-16 and miR-155 Highly significant differences were observed between the normal/stage 3 CKD and ESRD cohorts (miR-638, miR-21, miR-155, miR-210, miR-16) Significant differences were observed between Stage 4 CKD and ESRD patients for miR-210 and miR-16 The correlation between miRNA expression and eGFR remained significant when the patients undergoing haemodialysis are excluded from the analysis, with the exception of miR-21 and miR-155 levels
			Urine	22 CKD			No association between urinary miRNA level and kidney function Urine upregulation of miR-638 in stage 4 CKD compared to normal and stage 3 CKD patients (p < 0.006)
22045675	Starkey Lewis	2011	Serum	22 CKD 25 HC	Edinburgh	qRT-PCR: miR-122 miR-192	Overexpression of miR-122 and miR-192 in CKD patients

CKD: chronic kidney disease; CRP: C-reactive protein; eGFR: estimated glomerular filtration rate; ESRD: end-stage renal disease; HC: healthy controls; HD: haemodyalisis; HDL: high-density lipoproteins; IL-6: interleukin 6; miRNA(s): microRNA(s); NGS: Next-generation sequencing; PBMC: peripheral blood mononuclear cells; qRT-PCR: quantitative real-time polymerase chain reaction; RNA: ribonucleic acid; TGF-β: transforming growth factor beta

Recently, a panel composed of 16 miRNAs upregulated (let-7c-5p, miR-222–3p, miR-27a-3p, miR-27b-3p, miR-296-5p, miR-31-5p, miR-3687, miR-6769b-5p, and miR-877-3p) or downregulated (miR-133a, miR-133b, miR-15a-5p, miR-181a-5p, miR-34a-5p, miR-181c-5p, and miR1-2) has been identified in urine exosomes of 15 CKD patients compared to 10 healthy controls [124]. A similar 14 miRNAs panel (miR-29c-5p, miR-345-5p, miR-142-3p, miR-339-3p were upregulated and miR-17-5p, miR-130a-3p, miR-15b-5p, miR-106b-3p, miR-106a-5p, miR-16-5p, miR-181a-5p, miR-1285-3p, miR-15a-5p, miR-210-3p were downregulated) has been described in 15 African American CKD patients with treated hypertension compared to 15 controls (non-CKD patients with treated hypertension) [125]. In contrast with these results, downregulation of members of miR-29 and miR-200 was found in urine exosomes from 32 CKD patients when compared to 7 healthy controls [126].

Circulating miR-122 has been identified as a biomarker for ESRD, being substantially downregulated in 17 pre-haemodialysis patients compared to 22 healthy controls (19-fold lower), 30 patients with CKD (21.7-fold lower) and 15 transplanted patients [127]. Another biomarker differentially expressed in dialysis patients is miRNA-155, whose serum levels were found increased in 82 dialysis patients compared with 16 healthy subjects (P < 0.05) [128]. Interestingly, miR-155, along with other miRNAs involved in CVD, such as miR-21, miR-26b, or miR-146b, had been found differentially expressed in 10 clinically stable haemodialysis patients compared to 10 healthy controls [129]. In a larger study, circulating levels of miR-125b, miR-145 and miR-155 were found significantly decreased in 90 CKD and 10 haemodialysis patients compared to those in 8 healthy volunteers [130]. Decreased circulating levels of the three miRNA were also associated with progressive loss of eGFR [130].

The miRNA transcriptome has also been studied in platelets from five chronic haemodialysis patients and five stage 4 CKD uremic patients and compared with five age- and sex-matched healthy subjects with normal renal function [131]. Upregulation of miR-19b, involved in platelet reactivity, was found in uraemic CKD patients.

##### eGFR and CKD stratification

Total circulating small RNA level has been found decreased in 53 patients with impaired kidney function (CKD stages 3–5) compared to 22 controls (p < 0.0001, r = 0.553; Spearman rho) [132]. Furthermore, total small RNA concentration was threefold higher in plasma of normal/stage 3 patients compared to stage 4/ESRD (p < 0.0001). Specifically, the expressions of circulating miR-16, miR-21, miR-155, miR-210 and miR-638 were inversely correlated with eGFR [132]. Urinary miRNA level and kidney function was only associated for miR-638, which showed a significant increase in patients with stage 4 CKD compared to normal and stage 3 CKD patients (p < 0.006) [132]. In agreement with these results, decreased circulating levels of miR-125b, miR-145 and miR-155 were associated with progressive loss of eGFR by multivariate analyses in as study comprising 100 CKD and 10 haemodialysis patients of Caucasian and African American origin [130]. eGFR also inversely correlated with urinary level of miR-155 in 60 nephrolithiasis patients compared with 50 controls [133].

Another miRNA example associated with eGFR is miR-29c, which correlated with both eGFR (r = 0.362; p < 0.05) and degree of tubulointerstitial fibrosis (r = − 0.359; p < 0.05) in a group of 32 CKD patients [126].

More recently, 384 urinary and 266 circulatory miRNAs were found differentially expressed in 28 CKD patients when grouped by eGFR ≥ 30 vs. < 30 ml/min/1.73 m^2^ [134]. Among them, TGF-β signaling-related mRNA targets suggest that specific urinary and plasma miRNA profiles may act as diagnostic and prognostic biomarkers in CKD [134]. Urine downregulation of Let-7a and upregulation of miR-130 may identify eGFR < 30 ml/min/1.73 m^2^ patients, whereas upregulation of miR-1825 and miR-1281 in both urine and plasma, and plasma miR-423 downregulation would serve as an indicator of decreased eGFR [134].

#### Diabetic kidney disease

##### Prediction of DKD

Table 8 shows miRNA studies focused on DKD. The development of microalbuminuria was associated with a urinary miRNA signature composed of miRNAs known to be involved in the pathogenesis and progression of diabetic renal disease (miR-105-3p, miR-1972, miR-28-3p, miR-30b-3p, miR-363-3p, miR-424-5p, miR-486-5p, miR-495, miR-548o-3p and for women miR-192-5p, miR-720) in 17 T1DM patients exhibiting microalbuminuria compared to 10 T1DM without renal disease [135]. The same group had described a decrease in urine expression of miRNA-221-3p in a former study of T1DM patients with [10] or without DKD [10, 136]. In these patients, a panel of 10 miRNAs (underexpressed: miR-221-3p; overexpressed: miR-619, miR-486-3p, miR-335-5p, miR-552, miR-1912, miR-1224-3p, miR-424-5p, miR-141-3p, miR-29b-1-5p) was identified for patients with overt DKD [136]. Furthermore, downregulation of miR-323b-5p and upregulation of miR-122-5p was associated with persistent microalbuminuria. Appearance of microalbuminuria was associated with decreased miR-323b-5p and increased miR-429 urine levels [136]. Consistent with these findings, downregulation of miR-155 and miR-424, and upregulation of miR-130a and miR-145 was found in urinary exosomes from 12 microalbuminuric T1DM compared with 12 normoalbuminuric patients [137].

**Table 8:** miRNA studies in diabetic kidney disease

PMID	Author	Year	Sample	Patients	Ethnics	Methodology	Main findings
28667184	Cardenas-Gonzalez	2017	Urine Blood	Discovery cohort: 10 DKD, 10 HC Confirmation stage: 58 DKD, 119 HC Replication stage: 74 DKD, 71 DM, 30 HC	Caucasian	2402 microRNAs	Downregulation of miR-2861, miR-1915-3p and miR-4532 in patients with diabetic nephropathy, were (> 10-fold; p < 0.0001) Downregulation of miR-2861, miR-1915-3p and miR-4532 associated with eGFR (p < 0.01) and interstitial fibrosis/tubular atrophy (p < 0.05)
28923913	Liu	2017	Blood	T1DM/T2DM DKD/HC		RNA-Seq	Downregulation of serum miR-25 in patients with diabetes, both with and without nephropathy (p < 0.001)
26239688	Argyropoulos	2015	Urine	17 T1DM + mAlb 10 T1DM		qRT-PCR, Exiqon miRNA, qPCR panels 1 and 2	The development of microalbuminuria was associated with 18 differentially expressed microRNAs The predicted targets of these microRNAs map to biological pathways involved in the pathogenesis and progression of diabetic renal disease. A microRNA signature (miR-105-3p, miR-1972, miR-28-3p, miR-30b-3p, miR-363-3p, miR-424-5p, miR-486-5p, miR-495, miR-548o-3p and for women miR-192-5p, miR-720) achieved high internal validity for microalbuminuria
23358711	Argyropoulos	2013	Urine	10 DM-DKD 10 DM 10 DM + IMA 10 DM + PMA 10 HC		qRT-PCR miRNA panel	miRNA-221-3p expression decreased similarly to the comparison of follow up and baseline microalbuminuria samples miR-589 and miR-323b-5p showed a trend to be increased in the urine of patients with overt nephropathy Downregulation of miR-323b-5pand upregulation of: miR-122-5p, miR-429 in PMA patients Appearance of micro-albuminuria was associated with decreased levels of miR-323b-5p and increased urine concentration of miR-429
24223694	Barutta	2013	Urine Exosomes	12 T1DM + mAlb 12 T1DM + nAlb 10 non-diabetics		Human TaqMan miRNA Array A	Differential expression of miR-130a, miR-424, miR-155 and miR-145 in urinary exosomes from microalbuminuric T1DM Downregulation of miR-155 and miR-424 and upregulation of miR-130a and miR-145 in micro- compared to normoalbuminuric patients miR-155 and miR-424 could not discriminate between normoalbuminuric diabetic patients and non-diabetic controls Trend to overexpression of miR-145 and miR-130 in normoalbuminuric diabetic patients compared to controls
23797704	Fiorentino	2013	Tissue	8 DM + DKD 4 HC		qRT-PCR TaqMan miRNA reverse transcription kit	Overexpression of miR-21 in kidney biopsies from diabetic patients compared to HC Downregulation of miR-217 in diabetic subjects compared to HC
20056746	Krupa	2010	Biopsies	22 DKD	Caucasian	qRT-PCR TaqMan Low Density Array Human MicroRNA Panel 1.0	Two miRNAs differed by more than two-fold between progressors and nonprogressors, and 12 miRNAs differed between late presenters and other biopsies Late presenters showed the greatest decrease in miR-192 expression Downregulation of miR-192 correlated with tubulointerstitial fibrosis and low eGFR

DKD: diabetic kidney disease; DM: diabetes mellitus; eGFR:estimated glomerular filtration rate; HC: healthy controls; IMA: intermittent microalbuminuria; mAlb: microalbuminuria; MAlb: macroalbuminuria; nAlb: normoalbuminuria; PMA: persistent microalbuminuria; qRT-PCR: quantitative real-time polymerase chain reaction; RNA: ribonucleic acid; T1DM: type 1 diabetes mellitus; T2DM: type 2 diabetes mellitus

##### eGFR

In the largest miRNA study to date in DKD patients, downregulation of miR-2861, miR-1915-3p, and miR-4532 was associated with eGFR (p < 0.01) and interstitial fibrosis/tubular atrophy (p < 0.05) [138]. In a separate study, miR-192 downregulation was correlated with tubulointerstitial fibrosis and low eGFR in 22 DKD patients especially in those who had stage 5 CKD or required renal replacement therapy within 6 months of their renal biopsy [139].

The rate of eGFR decline positively correlated with the urinary miR-21 (r = 0.301; p = 0.026) and miR-216a (r = 0.515; p < 0.0001) in a combined analysis of patients with diverse CKD aetiologies including IgAN, diabetic glomerulosclerosis and hypertensive nephrosclerosis [140]. However, the separate analysis by disease only found urinary miR-216a levels correlated with the rate of eGFR decline in patients with hypertensive nephrosclerosis (r = 0.588; p = 0.005) and diabetic glomerulosclerosis (r = 0.605; p = 0.010) [140]. Other urinary miRNA biomarkers correlated with eGFR in IgAN patients are miR-200b (r = 0.512; p < 0.001) and miR-429 (r = 0.425; p = 0.005) [141]. In systemic lupus erythematous (SLE) patients, eGFR was correlated with urinary miR-146a in two studies from the same group (r = 0.242; p = 0.008) [142, 143]. Serum miR-146a also inversely correlated with proteinuria (r = − 0.341; p = 0.031) and the SLE Disease Activity Index (r = − 0.465; p = 0.003) in 40 SLE patients [142].

#### Primary glomerulonephritis

Although many studies have tried to find a specific signature of miRNAs in primary glomerulonephritis (Table 9), most of them are small sized cross-sectional designs which lack the statistical power and appropriate treatment of important issues, as correction for multiple testing, complicating the generalisation of their conclusions and limiting the extrapolation of their results.

**Table 9:** miRNA studies in primary glomerulonephritis

PMID	Author	Year	Sample	Patients	Ethnics	Methodology	Main findings
27000966	Duan	2016	Urine	Discovery cohort: 9 IgAN, 3 HC		Microarray Agilent human miRNA V19.0 chips (1888 human miRNA)	Differential expression of 214 miRNAs in the IgAN group (112 miRNAs with p < 0.01) Overexpression of miR-25-3p, miR-144-3p and miR-486-5p in IgAN (n = 93) compared to normal group (n = 82) or disease control (n = 40) miR-25-3p, miR-144-3p and miR-486-5p from urinary sediment were demonstrated to be mainly derived from urinary erythrocytes Overexpression of urinary supernatant microvesicles of miR-144-3p and miR-486-5p in the IgAN group
				Confirmation cohort: 30 IgAN, 20 HC		qRT-PCR: miR-25-3p miR-144-3p miR-486-5p miR-135a-3p miR-150-3p miR-638	Validation of four miRNAs (miR-25-3p, miR-144-3p-3p, miR-486-5p and miR-135a-3p) with p < 0.001
				Validation cohort: 63 IgAN, 62 HC		qRT-PCR: miR-25-3p miR-144-3p-3p miR-486-5p miR-135a-3p	Overexpression of miR-25-3p (p < 0.001), miR-144-3p (p = 0.040) and miR-486-5p (p < 0.001) in the IgAN group miR-135a-3p (p = 0.587) not differentially expressed Urinary sediment overexpression of miR-25-3p (p < 0.001), miR-144-3p (p = 0.046) and miR-486-5p (p = 0.010) in the IgAN group compared with disease control group The three urinary sediment miRNAs (miR-25-3p, miR-144-3p and miR-486-5p) showed good specificity and sensitivity for the diagnosis of IgAN (AUC value of 0.940 for the diagnosis of IgAN) Positive correlation of miR-144-3p level with the change in eGFR (r = 0.286; p = 0.020) and negatively correlated with the change in 24-h urine protein (r = -0.259; p = 0.019) Positive correlation of miR-25-3p level with the change in eGFR (r = 0.257; p = 0.038) Overexpression of miR-25-3p was associated with renal function improvement None of urinary sediment miRNA levels differed significantly between the groups with and without complete remission
26707063	Rudnicki	2016	Renal biopsy sections	Discovery cohort: 12 progressive, 31 stable		qPCR (miRNA) and microarrays (mRNA)	Downregulation of miR-30d, miR-140-3p, miR-532-3p, miR-194, miR-190, miR-204 and miR-206 in progressive cases Progressive cases also showed a downregulation of miR-532-3p
				Validation cohort: 8 progressive, 21 stable			Downregulation of miR-206 and miR-532-3p was confirmed
25682967	Ramezani	2015	Urine exosomes	16 FSGS 5 MCD 5 HC		GeneChip miRNA 3.0 Array qRT-PCR analysis	Downregulation of urinary levels of mir-1915 and miR-663 in patients with FSGS compared to MCD and controls (p < 0.001) Upregulation of urinary levels of miR-155 in patients with FSGS compared to patients with MCD and controls (p < 0.005) Inverse correlation of urinary levels of miR-663 with eGFR (r = -0.50; p = 0.04) in patients with FSGS Inverse correlation of urinary levels of miR- 1915 with proteinuria (r = 0.95; p = 0.003) Positive correlation of urinary levels of miR-663 with proteinuria (r = 0.45; p = 0.04)
			Plasma				Positive correlation of plasma levels of miR-342 and urine levels of miR-155 with proteinuria (r = 0.73; p = 0.02 and r = 0.72; p = 0.03, respectively) in patients with FSGS Upregulation of plasma levels of miR-30b, miR-30c, miR-34b, miR-34c and miR-342 and urine levels of mir-1225-5p in patients with MCD compared to patients with FSGS and controls (p < 0.001)
25218681	Zhang	2015	Plasma	Initial screening stage: 9 FSGS w/proteinuria, 9 HC		qRT-PCR	Overexpression of in miR-125b, miR-186, and miR-193a-3p levels were identified and confirmed in FSGS AUC of miR-125b, miR-186, miR-193a-3p, and the three miRNAs in combination were 0.882, 0.789, 0.910, and 0.963, respectively Downregulation of miR-125b and miR-186 concentrations in FSGS patients in complete remission compared to those with nephrotic proteinuria
				Confirmation stage: 32 FSGS w/proteinuria, 30 HC			
				37 FSGS w/proteinuria 35 CR FSGS			miR-125b and miR-186 levels declined markedly in patients with FSGS with complete remission, but not those with no response after steroid treatment. Plasma miR-125b and miR-186 levels were not elevated in patients with membranous nephropathy and diabetic nephropathy regardless of degree of proteinuria Plasma miR-186, but not miR-125b, level was correlated with degree of proteinuria in patients with FSGS (151 samples)
				Prospective: 51 FSGS w/proteinuria, 88 DC			
2.3E + 07	Wang	2013	Urine	20 DGS 21 MCN/FSGS 23 MGN 10 HC		qRT-PCR	Urinary miR-29a, miR-192 and miR-200c were not differentially expressed in patients with different causes of nephrotic syndrome Urinary sediment miR-29a, miR-192, and miR-200c levels were significantly different between diagnosis groups Downregulation of urinary miR-638 in all causes of nephrotic syndrome than HC Downregulation of urinary miR-192 in the DGS group Overexpression of urinary miR-200c in the MCN/FSGS group
22960330	Szeto	2012	Urine	17 IgAN 17 DGS 22 HTN	Asian	qRT-PCR	Downregulation of urinary miR-15 in DGS patients Overexpression of urinary miR-17 in patients with IgA nephropathy Trend to overexpression of urinary miR-216a and miR-21 in hypertensive nephrosclerosis
							Positive correlation of the rate of eGFR decline with the urinary expression of miR-21 (r = 0.301; p = 0.026) and miR-216a (r = 0.515; p < 0.0001) when all patients were pooled Separately, urinary miR-216a expression correlated with the rate of GFR decline in hypertensive nephrosclerosis (r = 0.588; p = 0.005) and diabetic glomerulosclerosis (r = 0.605, p = 0.010), but not IgA nephropathy (r = 0.042; p = 0.9). Urinary miR-21 expression did not correlate with the rate of eGFR decline for any diagnosis group Overexpression of urinary miR-21 and miR-216a was associated with dialysis-free survival in 18 patients who progressed to ESRD (log rank test, p = 0.005 and p = 0.003, respectively)
23272089	Wang	2012	Urine	10 FSGS	Asian	qRT-PCR	Overexpression of urine miR-10a and miR-30d in FSGS patients (≈ 13- and tenfold, respectively) Overexpression of urinary kidney-enriched miR-10a and miR-30d clearly indicated the kidney injuries in FSGS patients
22362909	Serino	2011	PBMC	Initial sample cohort: 25 IgAN, 25 HC	Italian	AgilentHuman miRNAMicroarraysV2/qRT-PCR	Thirty-seven miRNAs differentially expressed in PBMCs in IgA nephropathy Upregulation of miR-148b in IgA nephropathy
				Disease controls: 3 MPGN-I, 5 FSGS, 10 HSPN			
				Validation cohort: 50 IgAN, 50 HC			
23313053	Wang	2011	Urine	43 IgAN	Asian	qRT-PCR	Overexpression of both intra-renal and urinary of miR-146a and miR-155 IgAN Urinary levels of both miR-146a and miR-155 positively correlated with proteinuria but neither of them correlated with eGFR (r = 0.074 and r = -0.198, respectively)
20364043	Wang	2010	Urine	43 IgAN	Asian	qRT-PCR	Downregulation of urinary miR-200a, miR-200b and miR-429 in IgAN Proteinuria significantly correlated with urinary expression of miR-200a (r = -0.483; p < 0.001), miR-200b (r = -0.448, p = 0.001) and miR-429 (r = -0.466, p = 0.001) eGFR significantly correlated with urinary expression of miR-200b (r = 0.512; p < 0.001) and miR-429 (r = 0.425, p = 0.005), but not miR-200a (r = 0.246, p = 0.112). The rate of eGFR decline had a modest but significant correlation with urinary expression of miR-200b (r = 0.316, p = 0.041)

AUC: Area under the receiver operating characteristic curves; CR: complete remission; DC: disease controls; DGS: diabetic glomerulosclerosis; eGFR: estimated glomerular filtration rate; ESRD: end-stage renal disease; FSGS: focal segmental glomerulosclerosis; HC: healthy controls; HTN: hypertensive nephropathy; IgAN: IgA nephropathy; MCD: minimal change disease; MCN: minimal change nephropathy; MGN: membranous glomerulonephritis; miRNA(s) microRNA(s); mRNA: messenger ribonucleic acid; PBMC: peripheral blood mononuclear cells; qRT-PCR: quantitative real-time polymerase chain reaction

Overexpression of miR-25-3p, miR-144-3p or miR-486-5p in urinary erythrocytes was recently proposed as non-invasive diagnostic biomarkers for IgAN nephropathy in a study of 93 IgAN patients compared to 82 normal subjects and 40 disease controls [144].

Downregulation of miR-30d, miR-140-3p, miR-532-3p, miR-194, miR-190, miR-204 and miR-206 was associated with progression to ESRD or doubling of SCr in 43 patients with various glomerular diseases and decreased expression of miR-206 and miR-532-3p was confirmed in a validation cohort of 29 patients [123]. When the miRNA expression in the urinary sediment of 56 patients who had undergone kidney biopsy (17 diabetic glomerulosclerosis, 17 IgAN and 22 with hypertensive nephrosclerosis) was quantified, decreased miR-15 was associated with diabetic glomerulosclerosis, whereas increased miR-17 levels were shown in IgAN [140].

Other miRNA biomarkers proposed as indicators of IgAN are urinary and intra-renal overexpression of miR-146a and miR-155, described in 43 patients with IgAN compared to 13 healthy volunteers [145], urinary downregulation of miR-200a, miR-200b and miR-429 of patients with IgAN [141] and upregulation of miR-148b in PBMCs of patients with IgAN [146].

In 16 patients with FSGS, urinary downregulation of miR-1915 and miR-663 and miR-155 upregulation was found in patients when compared to five individuals with minimal change disease and five healthy controls (p < 0.001 and p < 0.005, respectively) [147]. Urinary levels of miR-663 were inversely correlated with eGFR (r = − 0.50; p < 0.04). Proteinuria correlated with higher plasma levels of miR-342 (r = 0.73; p = 0.02) and higher urine levels of miR-155 (r = 0.72; p = 0.03) and miR-663 (r = 0.45; p = 0.04), and with lower urinary levels of miR-1915 (r = 0.95, P = 0.003). These patients also showed upregulation of miR-30b, miR-30c, miR-34b, miR-34c and miR-342 in plasma and mir-1225-5p in urine [147]. In another study, an initial screening stage including plasma from nine FSGS patients and nine healthy controls identified a panel of three upregulated miRNAs (miR-125b, miR-186, and miR-193a-3p); the panel was subsequently confirmed in 32 FSGS patients and 30 healthy controls [148]. Increased plasma levels of miR-125b and miR-186 were also associated with nephrotic proteinuria [148]. Other proposed biomarkers for FSGS and kidney injury are miR-10a and miR-30d, which were found increased about 13- and 10-fold, respectively in the urine from 10 FSGS patients compared to 16 healthy controls [149].

#### Other kidney diseases

Table 10 shows miRNA studies developed in patients with other kidney diseases. Lupus disease activity has been associated with other biomarkers, such as miR-221 and miR-222, in the urinary sediment of 12 patients with LN [150]. These biomarkers have also been associated with macroalbuminuria in DKD patients [136] and CKD [124, 125]. Serum downregulation and urine upregulation of miR-146a and miR-155 was described in SLE patients compared to controls in two studies from the same group [142, 143]. Also in LN, downregulation of miR-3201 and miR-1273e (> threefold; p < 0.0001) and association with endocapillary glomerular inflammation was found in 89 LN patients (p < 0.01) [138].

**Table 10:** miRNA studies in other kidney diseases

PMID	Author	Year	Sample	Patients	Ethnics	Methodology	Main findings
28667184	Cárdenas-González	2017	Urine	Discovery cohort: 10 LN 10 HC Confirmation stage: 89 LN 119 HC Replication stage: 86 LN 37 SLE 30 HC	Caucasian	qRT-PCR miRNA in situ hybridisation 2402 urinary microRNAs	Downregulation of miR-3201 and miR-1273e (> threefold; p < 0.0001) in LN patients Association of miR-3201 and miR-1273e expression with endocapillary glomerular inflammation (p < 0.01)
28380212	Domenico	2017	Urine	41 graft dysfunction 8 stable patients	Brazil	qRT-PCR: miR-142-3p	Overexpression of urinary miR-142-3p in the acute tubular necrosis group (p < 0.001) and acute rejection group (p < 0.005) in comparison with stable patients Trend to higher urinary miR-142-3p expression, in the acute tubular necrosis group, compared with the acute rejection group (p = 0.079) ROC analysis: AUC was 0.77 (95%CI 0.62–0.92; p = 0.010). The cut-off selected was 2.03 and the resulting parameters were sensitivity = 92%; specificity = 87%; positive predictive value = 90% and a negative predictive value = 87% (p < 0.001)
			Blood				Overexpression of urinary miR-142-3p when acute tubular necrosis was compared with stable patients (p < 0.05) and with the acute rejection group (p < 0.05) No difference was found in the comparison between the stable patients and acute rejection groups (p = 0.921) ROC analysis: AUC was 0.75 (95%CI 0.56–0.94; p = 0.016). The cut-off selected was 1.59 and the resulting parameters were sensitivity = 75%; specificity = 63%; positive predictive value = 81%, and a negative predictive value = 50% (p < 0.05)
24489795	Ben-Dov	2014	Urine	20 CKD 20 ADPKD		barcoded adapter ligation, Illumina Genome Analyzer II	mir-143(2) members were 2.9-fold higher in urine cells from ADPKD compared to other CKD patients Expression levels of mir-133b(2) (4.9-fold) and mir-1(4) (4.4-fold) were lower in ADPKD
25197634	Hu Yang-Yang	2014	Serum	60 Nephrolithiasis, 50 HC	Asian	qRT-PCR: miR-155	The median levels of serum and urinary levels of miR-155 were significantly higher in nephrolithiasis patients than in controls eGFR inversely correlated with urinary level of miR-155
22685016	Guan	2012	Urine	12 SLE 10 NecGN		qRT-PCR	The degree of proteinuria significantly correlated with the urinary sediment level of miR-339-3P (r = 0.636; p < 0.001), but not other miRNA targets Urinary sediment miR-221 level correlated with serum C3 level (r = 0.658; p = 0.02). In the SLE group, urinary sediment miRNA levels did not correlate with the histological activity or chronicity index of the renal biopsy specimen
21987229	Wang	2012	Urine	40 SLE 13 HC	Asian	qRT-PCR: miR-146a and miR-155	Overexpression of urine miR-146a (2.27 [1.20–5.52] versus 0.87 [0.54–1.18]; p = 0.001) and miR-155 (2.28 [1.16–5.13] versus 0.99 [0.84–1.67]; p = 0.001) in SLE Correlation of eGFR with the level of urinary miR-146a (r = 0.242; p = 0.008), but not miR-155 (r = 0.069; p = 0.456)
20952466	Wang	2010		40 SLE 30 HC	Asian	qRT-PCR: miR-146a and miR-155	Downregulation of serum miR-146a and miR-155 levels, and upregulation of urinary level of miR-146a in SLE eGFR correlated with both serum miR-146a (r = 0.519; p = 0.001) and miR-155 (r = 0.384; p = 0.014) Serum miR-146a inversely correlated with proteinuria (r = − 0.341; p = 0.031) and the SLE Disease Activity Index (r = − 0.465; p = 0.003)

ADPKD: autosomal dominant polycystic kidney disease; C3: complement component 3; CKD: chronic kidney disease; eGFR: estimated glomerular filtration rate; HC: healthy controls; NecGN: pauci-immune necrotizing glomerulonephritis; LN: lupus nephritis; qRT-PCR: quantitative real-time polymerase chain reaction; RNA: ribonucleic acid; ROC: receiver operating characteristic; SLE: systemic lupus erythematous

Urinary overexpression of miR-142-3p, associated with CKD [125], has also been found in 41 kidney transplant recipients (23 patients with acute rejection and 18 with acute tubular necrosis when compared to eight stable patients (p < 0.001 and p < 0.005, respectively) [151]. Peripheral blood analysis in these patients also showed overexpression of miR-142-3p as a marker of acute tubular necrosis (p < 0.05 for both acute tubular necrosis/stable and acute tubular necrosis/acute rejection comparisons) [151].

Serum and urinary levels of miR-155 were found significantly higher in 60 nephrolithiasis patients compared with 50 controls [133]. miR-155 has also been associated with eGFR in CKD [130, 132, 133], proteinuria in FSGS [147], microalbuminuria in DKD [137], and proposed as diagnostic biomarker of CKD [129, 130, 148], FSGS [147] and IgAN [145].

### Conclusions

In the last decade, there have been major research efforts to find miRNA biomarkers capable of identifying and stratifying kidney diseases and associated phenotypic features, such as eGFR and proteinuria. Arguably the most promising miRNA biomarker associated with kidney diseases is miR-155, which is inversely correlated with eGFR, and found differentially expressed in CKD, IgAN, FSGS and nephrolithiasis. MiR-29 has also been found in several studies associated with CKD, overt albuminuria in DKD and nephrotic syndrome. However, the findings regarding whether its expression is up- or downregulated in CKD are conflicting, although these differences may be due to the sample source used, blood or urine exosomes. Other miRNA biomarkers proposed for kidney disease (miR-16, miR-17, miR-21, miR-30b, miR-122, miR-130, miR-142-3p, miR-146, miR-200, miR221, miR222, miR-486, miR-1915) have not been confirmed in more than two independent studies, indicating that further investigation needs to be done to clarify their role.

## Transcriptomics

Transcriptomics is the study of the complete set of RNA transcripts produced by the genome, in a specific cell or under certain circumstances [152]. The sum of RNA transcripts including mRNAs, ribosomal and transfer RNA, and regulatory noncoding RNAs comprise the transcriptome [153]. Throughout the past two decades, transcriptomics studies on renal disease have been carried out using different approaches, either studying the entire transcriptome or focusing on individual biomarkers. Since 1994, over 80 papers have tried to identify key regulators of different pathways which influence renal disease, including CKD, ESRD, DKD, Primary Glomerulonephritis, SLE and various other renal conditions. Most of the studies were performed on small numbers, with only three studies analysing results from over 100 patients (Table 11). A recent integrative bioinformatics analysis of 250 gene expression datasets of healthy renal tissues and those with various types of established CKD, including DKD, hypertensive nephropathy, and glomerulonephritis, retrieved from the Gene Expression Omnibus repository (https://www.ncbi.nlm.nih.gov/geo/) has identified nine genes significantly differentially expressed both in diseased glomeruli and tubules (*IFI16*, *COL3A1*, *ZFP36*, *NR4A3*, *DUSP1*, *FOSB*, *HBB*, *FN1*, *PTPRC*) [154]. Gene Ontology, pathway enrichment analysis and protein interaction network showed dysregulation of several metabolic, immune response, signalling pathways, platelet dysfunction, and extracellular matrix (ECM) organization in the glomeruli and signalling pathways in the tubules, mainly associated with apoptosis, PI3K-Akt, MAPK, and TGF-β [154].

**Table 11:** Transcriptomics studies in kidney diseases

PMID	Author	Year	Sample	Patients	Ethnics	Methodology	Main findings
29642064	Zhou	2018	Gene expression microarray datasets retrieved from GEO repository	18 DKD/40 HC, 26 HTN/44 HC, 68 IgAN/44 HC, 36 MGN/44 HC, 35 FSGS/62 HC	–	Integrative bioinformatics analysis Affymetrix U133 Plus 2.0 Affymetrix U133 A	Glomeruli: 176 genes were differentially expressed among all diseases, 104 upregulated (e.g., *FCN1*, *CX3CR1*) and 72 downregulated (e.g., *APOM*, *SLC13A1*). Gene Ontology analysis showed mainly cellular components associated with diseased glomeruli, as integral components of the plasma membrane, extracellular space, and within the MHC class II protein complex. Pathway enrichment analysis showed dysregulation of several metabolic, immune response, and signalling pathways (e.g., lipids/lipoproteins and amino acids metabolism, SLC-mediated transmembrane transport, and IFN-γ pathway). Protein interaction network identified a cluster mainly associated with platelet dysfunction, abnormal interleukin signalling, and extracellular matrix organization Tubules: 50 genes were differentially expressed among all diseases, nine upregulated (e.g., *COL3A1*, *MARCKS*) and 41 downregulated (e.g., *GDF15*, *RGS3*). Gene Ontology analysis showed association mainly with molecule binding and transcription factor activity. Pathway enrichment analysis showed dysregulation of several signalling pathways (e.g., cholecystokinin receptor (CCKR), apoptosis, PI3 K-Akt, MAPK, and TGF-β signalling pathways). Protein interaction network identified a cluster mainly associated with SMAD2/SMAD3/SMAD4 heterotrimer-regulated transcription, TGF-β receptor complex signalling, and interleukin signalling Nine genes were significantly differentially expressed both in diseased glomeruli and tubules (*IFI16*, *COL3A1*, *ZFP36*, *NR4A3*, *DUSP1*, *FOSB*, *HBB*, *FN1*, *PTPRC*)
28158872	Zhao	2017	CD4 + T Cells	15 SLE 15 HC	United States	qRT-PCR	TNFAIP3 expression has been found downregulated in CD4 + T cells from SLE
26986150	Zhai	2016	B lymphocytes	166 IgAN 77 HC	China	qRT-PCR	Increased *TNFSF13* expression is accompanied by upregulation of its receptors in B-lymphocytes from IgAN patients, inducing Gd-IgA1 overproduction
26631632	Ju	2015	Biopsy	55 CKD	United States	Microarray (Affymetrix GeneChip and TaqMan Low Density Arrays)	Urine levels of EGF correlated with tissue EGF transcript expression and predicted eGFR
26490557	Leal	2015	Peripheral Blood mononuclear cells	20 CKD 20 HD 11 HC	Brazil	qRT-PCR	*NFE2L2* mRNA was positively correlated with NF-κB mRNA expression in CKD patients (r = 0.52, p = 0.02) and healthy individuals
26116588	Perlman	2015	Blood	20 T2DM + DKD, 20 HC	United States	qRT-PCR	transcripts levels differentially expressed in T2DM with nephropathy elevated expression occurred in early stage 1-2, before a significant decline in eGFR Interest in *MCP*-*1*, *FGF*-*2*, *VEGF* and *EGF*
23921255	Hara	2013	Leukocytes	40 CKD 20 HC	Japan	qRT-PCR	*S100A12* expression was significantly higher in CKD patients compared to healthy controls
24347824	Yadav	2013	CD4^+^CD28^null^ peripheral mononuclear cells	25 CKD 8 HC	India	qRT-PCR	Basal expression of IFN-*γ*, perforin, and granzyme B in CD4^+^CD28^null^ cells was increased in CKD
23061738	Chen	2012	Urine	35 CKD 12 HC	China	qRT-PCR	*CTGF* and *NOV* genes were overexpressed in CKD
21906921	Spoto	2012	Plasma	75 CKD 33 HC	Italy	qRT-PCR	Upregulation of TNF-α was inversely correlated with eGFR Increase of plasma IL-6 in early stages of CKD, without showing any further increase at more severe stages
23024773	Rudnicki	2012	Biopsy	3 DKD 7 HTN 19 IgAN 10 MCD 7 FSGS 8 MGN 2 LN 2 MPGN 1 RPGN 16 Other	Switzerland	Microarray analysis	Correlation of versican isoforms V0 and V1 with creatinine levels
22969957	Tachaudomdach	2012	Biopsy	39 LN	Thailand	qRT-PCR	TGF-β1 and collagen I were correlated with *CTGF* expression Baseline eGFR and Renal *CTGF* mRNA expression were inversely correlated
20650904	Brabcova	2011	Biopsy	51 IgAN	Czech Republic	qRT-PCR	Higher TGF-β1 expression associated with IgAN progression
21640098	Chon	2011	Leukocytes	20 CKD 10 HC	Netherlands	qRT-PCR	Inverse correlation of leukocyte angiotensin II *AT1* receptor mRNA expression with renal function Leukocyte angiotensin II *AT1* receptor expression is higher in CKD
21040163	Lepenies	2010	Biopsy	64 CKD	UK	qRT-PCR	Inverse correlation of mRNA expression of peroxisome proliferator-activated receptor gamma (PPARγ) with renal function
19698090	Granata	2009	Peripheral blood mononuclear cells	9 CKD II-III, 17 HD, 8 HC	Helsinki	Microarray analysis	*COX6C*, *COX7C*, *ATP5ME*, and *UQCRH* were all significantly higher in CKD IV-V compared to CKD II-III and HC
18758661	Ibrahim	2008	Biopsy	20 T2DM, 4 normal tissue from renal tumor patients	Egyptian	qRT-PCR	Increased *PKC* alpha gene expression in diabetic patients with CKD *PKC* alpha gene concentrations and proteinuria had significant correlation in diabetic patients.
18784644	Zehnder	2008	Urine	174 CKD	81.6% white, 10.9% Asian, 6.9% Afro-Caribbean	qRT-PCR	*MCP*-*1* expression was increased in those with acute renal inflammation
16564935	Szeto	2006	Urine	131 CKD	China	qRT-PCR	Urinary *HGF* expression remained an independent predictor of the primary end point, it was increased in those at risk of end point.
15561743	Szeto	2005	Urine	29 CKD 10 HC	China	qRT-PCR	Overexpression of *TGF*-*β* and *MCP*-*1* was associated with glomerulosclerosis Overexpression of collagen IV was associated with CKD

Akt: Protein kinase B, also known as Akt; APOM: apolipoprotein M; AT1: angiotensin II Type 1 receptor; ATP5ME: ATP synthase membrane subunit e; CCKR: cholecystokinin receptor; CD4: cluster of differentiation 4; CD28: cluster of differentiation 28; CKD: chronic kidney disease; COL3A1: collagen type III alpha 1 chain; COX6C: cytochrome c oxidase subunit 6C; COX7C: cytochrome c oxidase subunit 7C; CTGF: connective tissue growth factor; CX3CR1: C-X3-C motif chemokine receptor 1; DKD: diabetic kidney disease; DUSP1: dual specificity phosphatase 1; EGF: epidermal growth factor; eGFR: estimated glomerular filtration rate; FCN1: ficolin 1; FGF: fibroblast growth factor; FN1: fibronectin 1; FOSB: FosB proto-oncogene; AP-1 transcription factor subunit; FSGS: focal segmental glomerulosclerosis; Gd-IgA1: galactose-deficient hinge region O-glycans; GDF15: growth differentiation factor 15; GEO: gene expression omnibus;, HBB: hemoglobin subunit beta; HC: healthy controls; HD: hemodialysis; HGF hepatocyte growth factor; HTN: hypertension; IFI16: interferon gamma inducible protein 16; IFN-γ: interferon gamma; IgAN: IgA nephropathy; IL-6: interleukin 6; LN: lupus nephritis; MAPK: Mitogen-activated protein kinases; MARCKS: myristoylated alanine rich protein kinase C substrate; MCD: minimal change disease; MHC: major histocompatibility complex; MCP-1: monocyte Chemoattractant Protein-1; MGN: membranous nephropathy; MPGN: membranoproliferative glomerulonephritis; mRNA: messenger ribonucleic acid; NF-κB: NF-kappa B complex subunits; NFE2L2: nuclear factor, erythroid 2 like 2; NOV: nephroblastoma overexpressed; NR4A3: nuclear receptor subfamily 4 group A member 3; PKC: protein kinase C; PPARγ: peroxisome proliferator-activated receptor gamma; PTPRC: protein tyrosine phosphatase, receptor type C; PI3K: phosphatidylinositol-3-kinases; qRT-PCR: quantitative reverse transcription polymerase chain reaction; RGS3: regulator of G protein signalling 3; RPGN: rapidly progressive glomerulonephritis; S100A12: S100 calcium binding protein A12; SLC: human solute carrier gene superfamily; SLC13A1: solute carrier family 13 member 1; SLE: systematic lupus erythematosus; SMAD2: SMAD family member 2; SMAD3: SMAD family member 3; SMAD4: SMAD family member 4; T1DM: type 1 diabetes mellitus; T2DM: type 2 diabetes mellitus; TNF: tumour necrosis factor superfamily; TGF-β1: transforming growth factor beta 1; TNF-α: tumour necrosis factor alpha; TNFAIP3: TNF alpha induced protein 3; TNFSF13: TNF superfamily member 13; TGF-β: transforming growth factor beta; UK: United Kingdom; UQCRH: ubiquinol-cytochrome c reductase hinge protein; VEGF: vascular endothelial growth factor; ZFP36: ZFP36 ring finger protein

### Chronic kidney disease

The association of mRNA expression profiles with the risk of CKD has been investigated in different such as human renal biopsies, urine or peripheral blood samples.

Urine samples were used to investigate the correlation of the mRNA expression of a panel of target genes with renal function in 29 CKD patients (12 IgAN and 17 glomerulosclerosis) and 10 healthy controls using qRT-PCR [155]. Overexpression of *TGF*-*β* and *MCP*-*1* was associated with glomerulosclerosis, and overexpression of collagen IV with both types of CKD. TGF-β1 plays an important role in the pathogenesis of CKD as it promotes inflammatory cell infiltration, tubular cell atrophy, mesangial cell hypertrophy and podocyte apoptosis [156], key features of CKD [157]. Urinary expression of connective tissue growth factor (CTGF) (r = − 0.471, p = 0.010) and collagen I (r = − 0.399, p = 0.032) were inversely correlated with the rate of eGFR decline after 12 months of follow-up, while TGF-β and MCP-1 did not have an influence on eGFR. CKD is characterized by the accumulation of ECM components in the glomeruli (glomerular fibrosis, glomerulosclerosis) and the tubular interstitium (tubulointerstitial fibrosis) [156]. TGF-β, IL-6 and MCP-1 expression was correlated to TLR4, which was significantly upregulated in human kidney biopsies from 70 CKD patients showing severe proteinuria and chronic ischaemic renal damage [158]. Both urinary and tissue MCP-1 also showed an increase in patients with acute renal inflammation in 174 patients with a variety of kidney diseases [159].

IL-6 expression was higher in 75 stage 2–5 CKD patients compared with 33 normal subjects, although it was not dependent on stage [160]. These CKD patients also showed upregulation of TNF-α, which was inversely correlated with eGFR [160].

The mRNA levels of *COX6C, COX7C, ATP5ME,* and *UQCRH* in peripheral blood mononuclear cells (PBMC) were all significantly higher in CKD stage 4–5 patients compared to nine CKD stage 2–3 patients and eight healthy controls [161].

Intrarenal mRNA expression of epidermal growth factor (*EGF*) in the tubulointerstitial compartment of kidney biopsies was identified as a potential predictive biomarker of eGFR at the time of the biopsy in 55 CKD patients [162].

Leukocyte angiotensin II AT1 receptor mRNA expression was inversely correlated with renal function in 20 CKD patients compared to 10 healthy subjects (r^2^ = 0.15, P < 0.03) [163].

Increased levels of versican expression positively correlated to histologic damage scores and to renal function in proteinuric kidney disease and impaired renal function in 74 renal biopsies from patients with various proteinuric kidney diseases. Versican isoforms V0 and V1 significantly correlated with SCr levels [164].

In 64 human kidney biopsies from CKD patients, mRNA expression of peroxisome proliferator-activated receptor gamma (*PPARγ*) correlated inversely with renal function [165]. In 35 CKD patients, urinary mRNA of *CTGF* and nephroblastoma overexpressed (*NOV*) gene were overexpressed compared to 12 healthy controls [166].

### End-stage renal disease

In 2006, Szeto analysed the role of urinary mRNA expression of 11 target genes as non-invasive markers on risk stratification of CKD patients in 131 patients with CKD followed until a primary endpoint of doubling SCr concentration or ESRD [155]. Urinary mRNA expression of hepatocyte growth factor was an independent predictor of the primary endpoint after adjustment for clinical and histological factors such as eGFR, gender and age and tubulointerstitial fibrosis [155].

Overexpression of IFN-*γ*, perforin, and granzyme B in CD4^+^CD28^null^ cells was associated with CKD in a study of 25 stage 4–5 CKD patients and 8 healthy subjects [167]. Higher expression of S100A12 in uraemic leukocytes has also been proposed as biomarker for ESRD in 40 stage 4–5 CKD patients compared to 20 healthy individuals (78.5 ± 70.5 vs. 23.7 ± 19.2 ng/ml; p = 0.0035) [168].

### Diabetic kidney disease

Diabetes, as one of the leading causes of ESRD [169], has also been the focus of transcriptomic biomarkers studies.

The role of two isoforms of protein kinase C (PKC)-alpha and beta, was investigated in renal biopsies using reverse transcription-PCR in 20 patients with T2DM. PKC-alpha gene expression was increased in diabetic patients with CKD [170].

Peripheral blood samples were analysed for expression of 35 gene transcripts. The results showed that serum MCP-1, FGF-2, VEGF, and EGF were all elevated at every stage of DKD. However, serum IL2RA was the only mediator to show a linear increase with the disease severity consistent with decreasing eGFR. The peripheral blood samples showed elevated levels of ICAM1, TNF-α, TGF-β, IL-8, IL17RA, IFNγ, and MYD88 at all stages of disease in this sample of 20 patients with clinical or biopsy confirmed DKD age, race and gender matched with healthy volunteers [169].

### Other renal diseases

Other CKD aetiologies, such as LN, have been investigated as potential beneficiaries of RNA expression biomarkers. *CTGF* mRNA expression was significantly correlated with TGF-β1, but inversely correlated with baseline eGFR, being higher in CKD stage 3–5 patients compared to stage 1–2 CKD in 39 patients with LN [171]. *CTGF* expression was positively associated with TGF-β1 [171].

Higher *TGF*-*β1* expression was suggested to be associated with IgAN progression, it was significantly associated with eGFR at the time of biopsy in 51 patients [172]. Upregulation of *TNFRSF13B* and *TNFRSF17* in B-lymphocytes appeared to be a trend in IgAN patients however statistical significance was not reached [173].

*TNFAIP3* expression has been found downregulated in CD4+ T cells from SLE patients compared to normal controls [174].

### Conclusion

Many studies have attempted to find transcriptomic biomarkers associated with a higher risk of CKD or biomarkers that predict changes in eGFR or proteinuria. However, most of the studies are limited in sample size and power and the biomarkers are not replicated in other studies. Expression of some cytokines as TGF-α and TGF-β1 and the matricellular protein CTGF have been proposed in different studies as potential biomarkers of renal function in CKD, but the evidence is not overwhelming.

## Concluding remarks

The proliferation of studies on omics-related biomarkers in the past decade reflects the need for novel, valid, non-invasive tools capable of identifying persons at risk of CKD and helping to target the management of renal disease. Identification of undiagnosed disease, stratification of patients and monitoring of kidney disease are fields that would benefit of such validated biomarkers.

Several genes, including *UMOD*, *SHROOM3* and *ELMO1* have been strongly associated with renal diseases, and some of their traits, such as eGFR and creatinine. The role of epigenetic and transcriptomic biomarkers in CKD and related diseases is still unclear. Multiple putative biomarkers have been identified but these are mainly derived from small, single centre studies. Confirmation of the utility of such biomarkers is needed in separate populations and in larger cohorts.

It is very unlikely that a single biomarker can be identified that can improve CKD risk prediction beyond current clinically used tests such as serum creatinine or urinary albumin excretion. Future progress in this area will come from combining multiple biomarkers into classifiers, including genomic, epigenomic, proteomic and/or metabolomic profiles, to allow more precision in the diagnosis of CKD and in assessing the prognosis for renal disease.

Screening for CKD by eGFR and/or albuminuria in high-risk populations, e.g. DM or hypertension has been suggested to be cost-effective, whereas screening in the general population is only cost-effective in certain situations, for instance older patients or those with rapid CKD progression who could be targeted for RAAS inhibitors for renal and cardiovascular risk reduction [175]. The use of complementary molecular biomarkers to help in the identification of patients with potentially progressive diseases, may delay the need of renal replacement therapy, a very expensive treatment which consumes a high percentage of healthcare budgets. An important factor to take into consideration is the small effect that individual-omic variants identified may have on complex traits, such as CKD and other kidney diseases, and consequently the elevated number of patients needed to test to obtain benefit. It will be necessary to integrate panels of several biomarkers to increase the prediction of CKD progression. Although the cost of conventional biomarkers is insignificant compared to molecular biomarkers, the construction of disease-customised chips for routine use may reduce the prices considerably and make them cost-effective, especially if they are designed to identify patients with a poor prognosis.

Genomics biomarkers face additional methodological challenges ahead, including complexity and cost-effectiveness, the establishment of adequate gold standards, standardisation of the different technologies and validation of the biomarkers in clinical trials, that need to be addressed properly before any clinical implementation can be incorporated into guidelines.

## Abbreviations

ACCS: 1-aminocyclopropane-1-carboxylate synthase homolog (inactive)
ADPKD: autosomal dominant polycystic kidney disease
APOL1: apolipoprotein L1
ATP5ME: ATP synthase membrane subunit e
CARe: Candidate-gene Association Resource Consortium
CARS: cysteinyl-tRNA synthetase
CD4: cluster of differentiation 4
CD28: cluster of differentiation 28
CE: capillary electrophoresis
CFHR1: complement factor H related 1
CFHR3: complement factor H related 3
CHARGE: Cohorts for Heart and Aging Research in Genomic Epidemiology Consortium
CKD: chronic kidney disease
CKDGen: Chronic Kidney Disease Genetics Consortium
COX6C: cytochrome c oxidase subunit 6C
COX7C: cytochrome c oxidase subunit 7C
CpGs: CpG islands or CG islands (5′-C-phosphate-G-3′: cytosine and guanine separated by only one phosphate group)
CTGF: connective tissue growth factor
CUBN: cubilin
CUX1: cut like homeobox 1
CVD: cardiovascular disease
DEFA: defensins, alpha
DKD: diabetic kidney disease
DM: diabetes mellitus
DNA: deoxyribonucleic acid
DNMTs: DNA methyltransferases
ECM: extracellular matrix
EGF: epidermal growth factor
eGFR: estimated glomerular filtration rate
eGFRcrea: estimated glomerular filtration rate based on creatinine levels
eGFRcys: estimated glomerular filtration rate based on cystatin c levels
ELMO1: engulfment and cell motility 1
EPO: erythropoietin
ESRD: end-stage renal disease
EWAS: epigenome-wide association studies
FGF: fibroblast growth factor
FHS: Framingham Heart Study
FRMD3: FERM domain containing 3
FSGS: focal segmental glomerulosclerosis
GENIE: GEnetics of Nephropathy an International Effort consortium
GPR39: G protein-coupled receptor 39
GWAS: genome-wide association study
HGF: hepatocyte growth factor
HDAC1: histone deacetylase 1
HLA: human leukocyte antigen complex
HLA-DPB1: major histocompatibility complex, class II, DP beta 1
HLA-DPB2: major histocompatibility complex, class II, DP beta 2
HLA-DQA1: major histocompatibility complex, class II, DQ alpha 1
HLA-DQB1: major histocompatibility complex, class II, DQ beta 1
HLA-DRB1: major histocompatibility complex, class II, DR beta 1
HORMAD2: HORMA domain containing 2
ICAM1: intercellular adhesion molecule 1
IFNγ: interferon gamma
IgAN IgA: glomerulonephritis
IL-6: interleukin 6
IL-8: interleukin 8
IL17RA: interleukin 17 receptor A
IL2RA: interleukin 2 receptor subunit alpha
ITGAM: integrin subunit alpha M
ITGAX: integrin subunit alpha X
KDIGO: Kidney Disease: Improving Global Outcomes
KDOQI: Kidney Disease Outcomes Quality Initiative
KL: klotho
KLF10: Kruppel like factor 10
LC–MS: liquid chromatography and mass spectrometry
LINE-1: long-interspersed nuclear element 1
LN: lupus nephritis
LUMA: luminometric methylation assay
MCD: minimal change disease
MCP-1: monocyte Chemoattractant Protein-1
Methyl-Seq: methylation sequencing
MGN: membranous glomerulonephritis
miRNA(s): microRNA(s)
mRNA: messenger RNA
MS: mass spectrometry
MTHFR: methylenetetrahydrofolate reductase
MYD88: myeloid differentiation primary response 88, innate immune signal transduction adaptor
MYH9: myosin heavy chain type II isoform A
NKF: National Kidney Foundation
NOV: nephroblastoma overexpressed
ODF1: outer dense fiber of sperm tails 1
PHB: prohibitin
PKC: protein kinase C
PKD1: polycystin 1, transient receptor potential channel interacting
PKD2: polycystin 2, transient receptor potential cation channel
PLA2R1: phospholipase A2 receptor 1
PBMC: peripheral blood mononuclear cells
PPARγ: peroxisome proliferator-activated receptor gamma
PRKAG2: protein kinase AMP-activated non-catalytic subunit gamma 2
PRKCE: protein kinase C epsilon
PSMB9: proteasome subunit beta 9
PTPRN2: protein tyrosine phosphatase, receptor type N2
RNA: ribonucleic acid
ROC: receiver operator characteristics curve
ROS: reactive oxygen species
RUNX3: runt related transcription factor 3
SALL1: spalt like transcription factor 1
SCr: serum creatinine
SHC1: Src Homologous and Collagen adaptor protein 1
SHIP: Study of Health in Pomerania
SHROOM3: shroom family member 3
SLC22A2: solute carrier family 22 member 2
SLC43A2: solute carrier family 43 member 2
SLE: systemic lupus erythematosus
SMAD6: SMAD family member 6
SNP(s): single nucleotide polymorphism(s)
ST3GAL5: ST3 beta-galactoside alpha-2,3-sialyltransferase 5
ST6GAL1: ST6 beta-galactoside alpha-2,6-sialyltransferase 1
STC1: stanniocalcin 1
STK24: serine/threonine kinase 24
T1DM: type 1 diabetes mellitus
T2DM: type 2 diabetes mellitus
TAP2: transporter 2, ATP binding cassette subfamily B member
TGFβ: transforming growth factor beta
TGFBR3: transforming growth factor beta receptor 3
TNF-α: tumour necrosis factor alpha
TNFAIP3: TNF alpha induced protein 3
TNFRSF13B: TNF receptor superfamily member 13B
TNFRSF17: TNF receptor superfamily member 17
UACR: urinary albumin-to-creatinine ratio
UAE: urinary albumin excretion
UMOD: uromodulin
UMODL1: uromodulin like 1
UMODL1-AS1: UMODL1 antisense RNA 1
UQCRH: ubiquinol-cytochrome c reductase hinge protein
VEGF: vascular endothelial growth factor
WDR37: WD repeat domain 37
WHSC1: Wolf-Hirschhorn syndrome candidate 1
